# Therapy after therapy: Entry rates into subsequent psychological treatment among patients receiving therapist‐guided internet‐delivered or face‐to‐face psychotherapy

**DOI:** 10.1111/bjc.70036

**Published:** 2026-01-05

**Authors:** A. Plattonen, S. Mylläri, S. E. Saarni, T. Rosenström

**Affiliations:** ^1^ Department of Psychology Faculty of Medicine, University of Helsinki Helsinki Finland; ^2^ Department of Psychiatry Helsinki University Hospital Helsinki Finland; ^3^ Department of Psychiatry Faculty of Medicine and Health Technology, Tampere University Tampere Finland; ^4^ Department of Psychiatry Wellbeing Services County of Päijät‐Häme Lahti Finland

**Keywords:** mental health care services, psychological treatments, register study, therapist‐guided iCBT

## Abstract

**Objectives:**

Despite numerous psychotherapy trials, knowledge on service‐system efficiency in terms of return to treatment is limited, especially regarding internet‐delivered cognitive behavioural therapy (iCBT). We estimated the hazard ratio (HR) of subsequent psychological treatment over several years following the initial psychological intervention.

**Methods:**

This naturalistic register follow‐up study in Finland included patients receiving therapist‐guided iCBT (2013–2021, *n* = 30,934) or ≤20‐session psychotherapy (2018–2021, *n* = 3348), and matched population controls (*n* = 92,846). Their long‐term psychotherapy data (≤200 sessions/3 years, requiring prior treatment) were obtained from the Social Insurance Institution. We used Cox proportional hazard regression, adjusting for age, sex, first purchase of psychotropic drugs and onset of the first psychiatric diagnosis.

**Results:**

Given the adjustments, the hazard of subsequent long‐term psychotherapy was fourfold after iCBT (HR = 4.08; 95% CI 3.81–4.37) and nearly ninefold after ≤20‐session psychotherapy (HR = 8.94; CI 7.79–10.26), compared to those without these prior treatments. Prior ≤20‐session psychotherapy was associated with reduced hazard of entering subsequent iCBT (HR .12, CI .09–.16), while prior long‐term psychotherapy was not (*p* = .087). Prior iCBT was associated with a lower hazard of subsequent ≤20‐session psychotherapy (HR = .41, CI = .35–.47), while prior long‐term psychotherapy was not (*p* = .332).

**Conclusions:**

Mapping the succession of psychological treatments added knowledge and revealed surprises. For example, patients receiving therapist‐guided iCBT were less likely to access subsequent face‐to‐face psychotherapy than those initially treated face‐to‐face. While past services are used as a convenience indicator for future services, future research on successive psychological treatments should continue to disentangle clinical need from service systems effects.

**Practitioner points**

- While evidence on return to treatment after psychological interventions remains limited, our large‐scale register‐based observational follow‐up study provided the first real‐world estimate of subsequent treatment entry rate after therapist‐guided internet‐delivered therapy.
- In Finnish routine care, patients receiving therapist‐guided iCBT were less likely to access subsequent face‐to‐face psychotherapy than those initially treated face‐to‐face.
- Immediate treatment response to guided iCBT or ≤20‐session psychotherapy did not consistently and reliably predict whether patients entered subsequent treatment.
- Deepening our understanding of treatment sufficiency is crucial for service‐system development. However, since both individual and system‐level factors in mental health services affect subsequent treatment use, further research should continue to disentangle these factors.

## INTRODUCTION

Need for psychosocial treatments exceeds supply (Santomauro et al., 2024; Thornicroft et al., 2017; Vigo et al., 2020). To address this, many countries are shifting towards shorter‐term and internet‐delivered therapies, which require less therapist time and training, allowing higher patient volumes (Baigent et al., 2023; Knapstad et al., 2018; Kobori et al., 2014; S. I. Saarni et al., 2022; Wakefield et al., 2021). While therapist‐guided internet‐delivered therapies are a promising tool for scaling up treatment provision, producing treatment effects comparable to face‐to‐face CBT (Cuijpers et al., 2010; Hedman‐Lagerlöf et al., 2023; Rosenström et al., 2025), seeking treatment after iCBT remains largely undocumented (Andersson et al., 2018; Mamukashvili‐Delau et al., 2023). Symptom reduction alone may not ensure cost‐effectiveness if multiple subsequent treatments are used. On the other hand, offering more treatment has not always reduced disorder prevalence (see treatment–prevalence paradox, Ormel et al., 2022), and more extensive therapies may not be more cost‐effective than short‐term ones despite the additional treatment use observed with shorter treatments (Maljanen et al., 2016). Even though the prevalence of mental disorders might be partially reduced by interventions, the likelihood of developing a new disorder after receiving treatment remains high (Menzies et al., 2024). Hence, to improve service systems, comprehensive real‐world data on subsequent treatment use across interventions delivered at various levels of stepped or stratified care (Delgadillo et al., 2022) are needed.

Recent meta‐analyses emphasize the importance of psychological interventions for sustainable treatment effects for depression (Bruin et al., 2022; Cuijpers et al., 2023; Furukawa et al., 2021; Levy et al., 2021). Still, absolute response rates to psychotherapies are modest, as most patients do not achieve a ≥50% reduction in symptoms, with such response rates ranging between .24 and .42 for eight major mental disorders (Cuijpers et al., 2024). While residual symptoms predict relapse across treatment modalities (Buckman et al., 2018; Delgadillo et al., 2018; Palacios et al., 2022; Wojnarowski et al., 2019), evidence on actual return to treatment is scarce. Symptom recovery may have little association with treatment return (Lorimer et al., 2024), and in some cases, its interaction with high therapy attendance may even increase return to treatment (Reeder et al., 2020).

It remains unclear whether any psychological treatment slows the ‘revolving door’ in mental health services, referring to repeated episodes of mental disorders and treatments (Menzies et al., 2024). In randomized studies, additional treatment seeking has been common, reported in about 60% of participants receiving short‐ or long‐term face‐to‐face therapy (Knekt et al., 2011) and in 55% of those receiving internet‐delivered therapy (Andersson et al., 2013). Only a few studies have estimated return rates after psychological treatment in routine services. In the UK, 13.7% of patients accessed further treatment within 1–5 years in the same NHS Talking Therapies service (Lorimer et al., 2024). Comparably, in the Netherlands, 14% of patients returned to the same service for further treatment within 1–3 years (Boerema et al., 2016). The observed association between treatment duration and return rates has been weak or negligible (Boerema et al., 2016; Lorimer et al., 2024). Furthermore, return rates to the same treatment provider leave out an important group of dissatisfied patients who seek further treatment solutions from other providers or treatment categories. The quality of evidence is weak regarding transitions across different service providers. For example, only one study has reported return to treatment following internet‐delivered therapies (Andersson et al., 2013).

**FIGURE 1:**
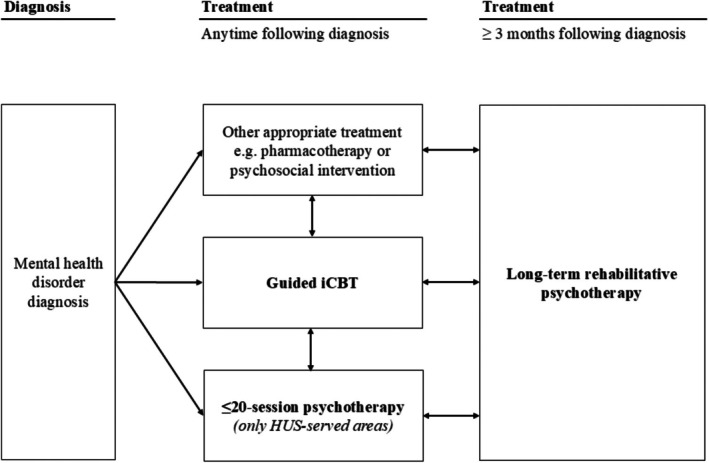
Overview of potential subsequent treatment pathways following a mental health disorder diagnosis in the Finnish mental health care system. Entry rates into the bolded treatments were estimated in this study.

We aimed to address this research gap by estimating the entry rates into subsequent psychological treatment among patients who had received guided iCBT or face‐to‐face psychotherapy in Finnish routine care (see Figure 1 and Table 1). Therapist‐guided iCBTs for specific mental disorders have been part of Finnish public health care since 2013 (Rosenström et al., 2023), with 12,800 patients treated nationwide in 2021. While the offering of shorter face‐to‐face psychotherapies has varied across regions, the most prevalent form of psychotherapy has been very long‐term rehabilitative psychotherapy (up to 200 sessions in 3 years) with 60,800 annual recipients in 2021 (Social Insurance Institution of Finland, 2022). Aimed at improving clients' ability to work and study, long‐term psychotherapy is financially supported by the Social Insurance Institution. However, it requires partial self‐payment by the client and is contingent on 3 months of prior adequate treatment (e.g. iCBT, short‐term psychotherapy, other psychosocial interventions or pharmacotherapy).

**TABLE 1:** Descriptions of the treatments studied.

	Guided iCBT	20‐session psychotherapy	Long‐term rehabilitative psychotherapy
Organizer	HUS Helsinki University Hospital	HUS Helsinki University Hospital	Social Insurance Institution of Finland
Available since	2013^a^	2016	2011^b^
Data available since	2013	2018^c^	2011
Length	5–12 weekly sessions regarding the specific programme	Up to 20 sessions	Up to 200 sessions in 3 years
Form	Independent work in specific programme targeting depression, generalized anxiety, insomnia, social anxiety (adult and adolescent version), obsessive‐compulsive disorder, panic disorder, bipolar disorder, bulimia, harmful alcohol use or unexplained somatic symptoms	Individual, parent‐infant, group, family or couples' psychotherapy either live or remotely	Individual, group, family or couples' psychotherapy and arts therapy live or remotely
Psychotherapeutic framework	Cognitive behavioural therapy	Various (i.e. cognitive, cognitive behavioural, psychodynamic, solution‐focused, integrative and family therapy)	Various (i.e. cognitive, cognitive behavioural, psychodynamic, solution‐focused, integrative and family therapy)
Therapist	Clinical psychologist or nurse	Licensed psychotherapist	Licensed psychotherapist
Selection of therapist	Patients are referred to a therapist	Patients select their psychotherapist from approved private providers	Patients select their psychotherapist from approved private providers
Inclusion and prerequisitions	Patients with mild to moderate psychiatric symptoms, access to Internet, email and phone number required	Patients with mental health disorders that can be treated with psychotherapy	(1) A person's ability to work/study is impaired by a mental disorder AND (2) The person has received at least 3 months of appropriate treatment post‐diagnosis AND (3) A psychiatrist deems the therapy necessary to improve the person's ability to work or study
Eligible age range	≥ 16 years	≥18 years^d^	16–67 years
Exclusion	Vary slightly between programmes, yet common for all are severe suicidality, acute psychosis, severe personality disorder or cognitive decline that interferes with treatment	Current severe suicidality, psychosis, severe undernourishment due to an eating disorder, antisocial behaviour or substance abuse	Permanently outside of working life, difficulties in coping with everyday life due to illness severity
Fee for a patient	Free	Free	Fixed financial support, typically covering approximately half to two‐thirds of the client's costs^e^, psychotherapists deem their own fee and patient pays the separation
Referrals	Any doctor in Finland including public, occupational and private sectors	Public primary care or psychiatric specialist care in HUS‐served areas	Psychiatrist either public, occupational or private sector
Multiple access	Not restricted	If targeted to different mental health disorder	Previous rehabilitative psychotherapy concluded ≥5 years ago
Coverage	Nationwide	HUS‐served areas excluding Helsinki, 19% of Finnish population	Nationwide

^a^ First HUS‐iCBT programme (depression) launched in May 2013.

^b^ Rehabilitative psychotherapy changed from discretionary to mandatory statutory right in Finland in 2011.

^c^ Finnish Psychotherapy Quality Register for adult patients was launched in September 2018 (FPQR; Saarni et al., 2023).

^d^ ≤20‐sessions outsourced psychotherapies are provided for adolescents and children, but not included in this study.

^e^ Compensations vary between therapy forms and are currently 57.60€ per session for individual therapy.

In this paper, we estimated (i) whether receiving therapist‐guided iCBT or up to 20‐session psychotherapy is associated with entry rates to subsequent long‐term psychotherapy and, more generally, (ii) how these treatments are associated with treatment entry rates to each other. Additionally, we explored (iii) whether these associations changed from 2018 to 2021, given the increase in the provision and utilization of psychological treatments in Finland. Since therapist‐guided iCBT and face‐to‐face therapy have produced comparable treatment outcomes (Cuijpers et al., 2010; Hedman‐Lagerlöf et al., 2023; Rosenström et al., 2025), we hypothesized no significant difference in subsequent treatment entry rates between these delivery methods.

## METHODS

### Sample and procedure

The participants of this naturalistic register follow‐up study included patients from hospital treatment registries—the HUS Helsinki University Hospital iCBT register (HUS‐iCBT), which includes the nationwide iCBT service, and the Finnish Psychotherapy Quality Register (FPQR; Saarni et al., 2023), which covers outsourced psychotherapies within the HUS‐served area—and their matched population controls. The seed sample included a complete cohort of patients who had been referred to HUS‐provided iCBT (2013–2021) or outsourced psychotherapy of up to 20 sessions (2018–2021). First, for every patient, two controls matched for age, gender and place of residence were collected from the Population Registry of the Digital and Population Data Services Agency. Then, pseudonymized register data were collected for each patient and control by various registry holders using social security identification numbers to link between registers. Information on the use of long‐term rehabilitative psychotherapy (up to 200 sessions over 3 years) and prescription‐drug purchases was obtained from the Social Insurance Institution of Finland, while public‐sector diagnoses were sourced from the Care Register for Health Care of the Finnish Institute for Health and Welfare. Our research permission was granted by the Finnish Social and Health Data Permit Authority Findata (THL/4810/14.02.00/2020 and THL/1303/14.06.00/2023). As the study used only register data, no informed consent was required according to Finnish laws. The study was approved by the Ethical Committee of HUS (HUS/3150/2020).

**FIGURE 2:**
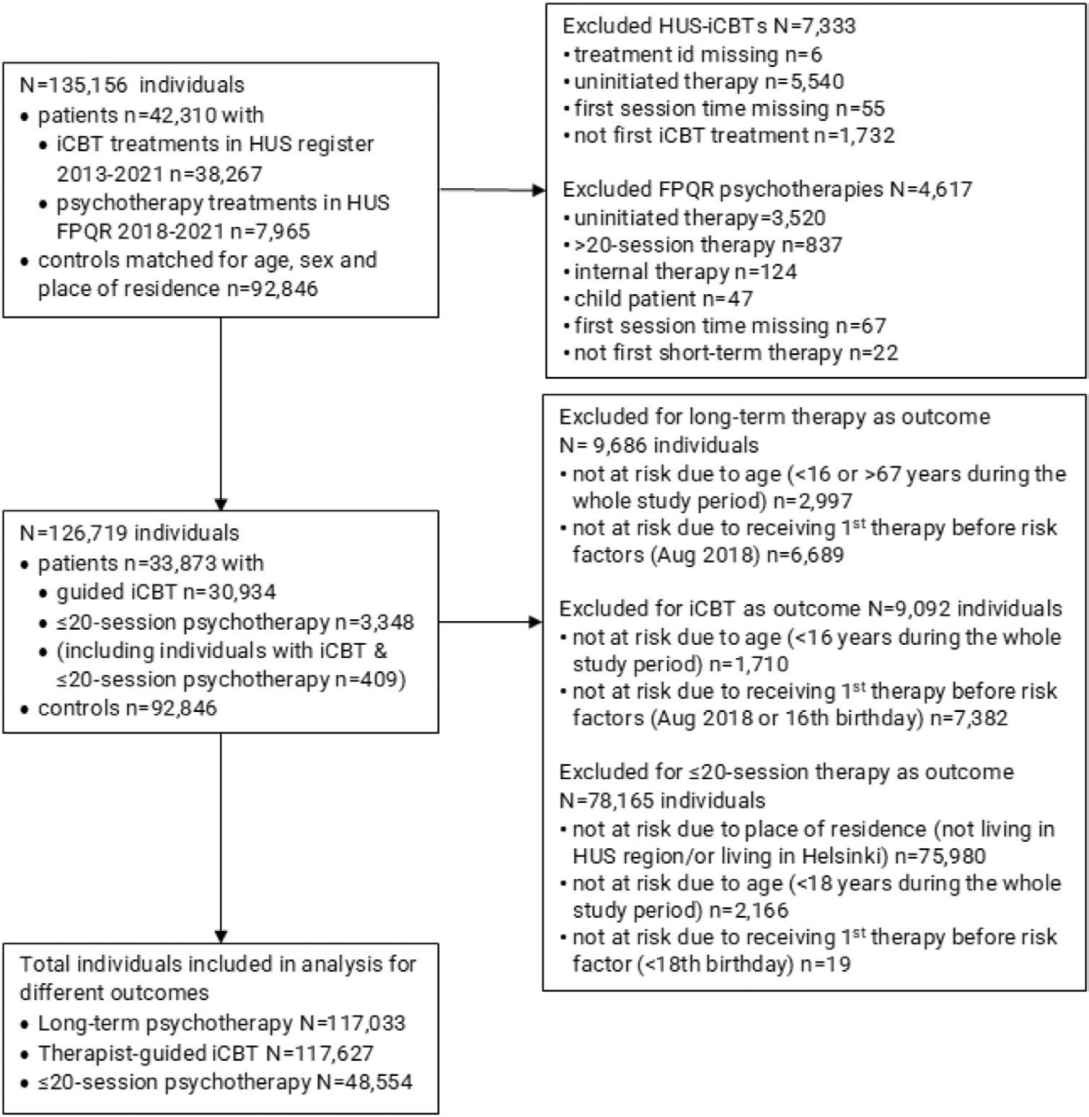
Flowchart of the participants included in the study and their inclusion in or exclusion from the different outcome analyses.

Our original sample included 135,156 individuals (Figure 2). Patients could have received multiple episodes of iCBTs and/or ≤20‐session psychotherapies; however, only the first treatment of each type was included in the analysis to focus on treatment pathways between distinct treatment types. Controls received neither of these two treatments. However, both patients and controls could have received long‐term psychotherapy, and only the first treatment episode was considered. For detailed descriptions of the psychological treatments studied and the possible pathways between subsequent treatments, see Figure 1 and Table 1.

Data on long‐term psychotherapy were available from January 2011, on guided iCBT from May 2013 (launch of the first HUS‐iCBT programme), and on ≤20‐session psychotherapy from August 2018 (implementation of FPQR; Saarni et al., 2023) to the end of 2021 for all treatments (Table 1).

### Treatment entry

The main outcome variable was the time from the start of follow‐up to possible subsequent treatment entry. For long‐term psychotherapy, treatment entry was defined as the first date of financial reimbursement decision. For guided iCBT and ≤20‐session psychotherapy, it was the date of the first baseline questionnaire.

### Covariates

Prior guided iCBT, ≤20‐session psychotherapy or long‐term psychotherapy, excluding the outcome treatment of each model, were used as a time‐dependent covariate. Covariate status changed to ‘treated’ at the initiation of the respective prior treatment and remained so for the rest of the follow‐up period. In addition to prior psychological treatment, we adjusted for several variables related to the likelihood of mental health treatment (Roberts et al., 2018; see Table S1 for covariate rationale). The patient's age at the time of their first record in the HUS‐iCBT register or FPQR (or for controls, the age at the time of index patient's first record) and sex (female and male) were baseline covariates.

In addition, we used the following time‐dependent covariates. Our data included all psychiatric diagnoses (F codes in the International Classification of Diseases, 10th edition (ICD‐10)) given in public speciality care, but excluded those given in primary, private or occupational health care. To avoid over‐adjusting treatment pathways, we used only diagnoses given before any psychological treatment studied. Additionally, we included the date of the first purchase of any psychotropic drugs. The first purchase date of a paracetamol prescription served as a negative control.

To adjust for symptom change during guided iCBT and ≤20‐session psychotherapy, we used the target symptom score of each treatment (see Section S2 and Table S2). Symptom change was the difference in the symptom scores from the beginning of the treatment to the last observed measurement, standardized within each treatment. We used each patient's last observed measurement because it reflects the actual change demonstrated by the patient in real‐world clinical settings, where non‐adherence, early termination and dropouts are common. This approach prioritizes ecological validity over idealized assumptions of complete treatment adherence. As a sensitivity analysis, we applied multiple imputation to address missing symptom scores in therapist‐guided iCBT and ≤20‐session psychotherapy, including both completely missing values and cases where the last available symptom observations were prior to treatment‐programme end. This approach strives to estimate the treatment's potential efficacy under ideal, though unrealistic, conditions (see Section S4). Symptom data for long‐term psychotherapy are not systematically collected and were therefore unavailable.

### Statistical analyses

We used R version 4.2.2, with packages ‘survival’ version 3.4–0 and ‘survPen’ version 1.6.0. The Cox proportional hazards model (Cox, 1972) was used to regress the risk of treatment entry (‘event’) on the time‐dependent covariate of prior initiation of another treatment (either guided iCBT, ≤20‐session psychotherapy or long‐term psychotherapy, excluding the outcome treatment). We performed univariate analyses for each prior treatment separately, followed by a multivariate analysis to adjust for covariates and the interaction of prior treatments. The hazard ratio (HR) indicates the change in entry rate into the outcome treatment after a risk‐factor onset.

We estimated temporal associations between all ordered treatment pairs including the first possible treatment of each type. Follow‐up began when individuals became at risk for the outcome event and psychological covariate treatment was available. Accordingly, follow‐up started at the earliest on 25 August 2018; the individual's 16th birthday for iCBT or long‐term psychotherapy; or their 18th birthday for ≤20‐session psychotherapy. Data were available until the end of 2021. Follow‐up was terminated at the entry into the outcome intervention or on 30 June 2021 (whichever came first) to avoid an artificial decline in intervention use due to potential sampling issues at the end of the data collection period. For long‐term psychotherapy aimed at supporting employability, follow‐up was alternatively ended at retirement age, 68. Days were used as the unit of time.

We estimated baseline hazards with 95% confidence intervals for different treatment entries. We also tested the proportional hazard assumption (Grambsch & Therneau, 1994) for each time‐dependent psychological treatment covariate using the ‘cox.zph’ function from the survival package to diagnose non‐proportional hazards. The Cox proportional hazards model, being semi‐parametric, inherently accounts for the changes in base rates over time. Thus, although our follow‐up period includes years affected by the COVID‐19 pandemic, the model accounts for the temporal shifts in base rates, including those related to the pandemic. For supplementary analyses specifically addressing the impact of COVID‐19 and data missingness (van Buuren & Groothuis‐Oudshoorn, 2011), see Sections S4, S5.

## RESULTS

### Sample characteristics

This study included 126,719 unique individuals. Table 2 presents the characteristics of the controls and participants who received any of the psychological treatments by the end of 2021. An individual can receive one or many treatments. Without accounting for treatment sequence (which is analysed below), 1.3% of guided iCBT patients entered also to ≤20‐session psychotherapy**—**though regional restrictions meant not all were eligible (only the eligible region is analysed below). Of the guided iCBT patients, 22.2% received long‐term psychotherapy at some point during their follow‐up. Among those who received ≤20‐session psychotherapy, 12.2% received also guided iCBT and 17.9% long‐term psychotherapy.

**TABLE 2:** Participants background variables for each treatment (*n* = 126,719 unique individuals, same person can receive many treatments).

	No treatment	Therapist‐guided iCBT	≤20‐session psychotherapy	Long‐term psychotherapy
*N*	87,678	30,934	3348	12,566
Sample overlap (*n*)				
No treatment	87,678	0	0	0
Therapist‐guided iCBT	0	30,934	409	6880
≤20‐session psychotherapy	0	409	3348	600
Long‐term psychotherapy	0	6880	600	12,566
Demographics				
Female, *n* (%)	60,572 (69.08)	22,038 (71.24)	2645 (79.00)	10,510 (83.64)
Age at first register onset^a^ mean (SD)	34.09 (12.91)	34.36 (12.25)	36.88 (14.82)	33.11 (9.81)
Mental health				
At least one psychiatric diagnosis^b^, *n* (%)	17,728 (20.22)	22,122 (71.51)	2255 (67.35)	9115 (72.54)
Mean number of psychiatric diagnoses (SD)	.72 (2.29)	2.33 (3.40)	2.52 (3.49)	2.87 (3.81)
Most frequent psychiatric diagnosis^b^, *n* (%)				
Depressive disorder	4296 (4.90)	8295 (26.82)	840 (25.09)	3496 (27.82)
Anxiety disorder	4743 (5.41)	8992 (29.07)	918 (27.42)	3608 (28.71)
Alcohol‐related disorder	1293 (1.47)	613 (1.98)	50 (1.49)	168 (1.34)
Physiology‐related mental disorder	1196 (1.36)	1481 (4.79)	124 (3.70)	645 (5.13)
Psychotic or bipolar disorder	722 (.82)	470 (1.52)	43 (1.28)	231 (1.84)
Other diagnosis	5436 (6.20)	2244 (7.25)	278 (8.30)	955 (7.60)
No psychiatric diagnosis^b^	69,992 (79.83)	8839 (28.57)	1095 (32.72)	3463 (27.56)
Treatment received, *n* (%)				
>1 psychological treatment^c^	0	7207 (23.30)	927 (27.69)	7398 (58.87)
Therapist‐guided iCBT	0	30,934 (100)	409 (12.22)	6880 (54.75)
≤20‐session psychotherapy	0	409 (1.32)	3348 (100)	600 (4.77)
Long‐term psychotherapy	0	6880 (22.24)	600 (17.92)	12,566 (100)
At least one purchase of psychotropic drugs	25,116 (28.65)	26,796 (86.62)	1387 (41.43)	10,683 (85.02)

*Note*: Data include all treatment records available up to the end of 2021. For specific analyses, only individuals in the relevant risk set and with treatments initiated before July 2021 were included (see Figure 2).

^a^ Patient's age at first HUS‐iCBT/FPQR record; for controls, age at index patient's first record.

^b^ Psychiatric diagnoses (ICD‐10) given in public speciality care categorized as: depression (F32‐F39), anxiety (F40‐F48), physiology‐related disorders (F50‐F59), psychotic or bipolar (F20‐F31), alcohol‐related (F10) or other diagnoses (all other F diagnoses).

^c^ Including therapist‐guided iCBT, ≤20‐session psychotherapy or long‐term psychotherapy.

### Baseline hazards of different treatments

**FIGURE 3:**
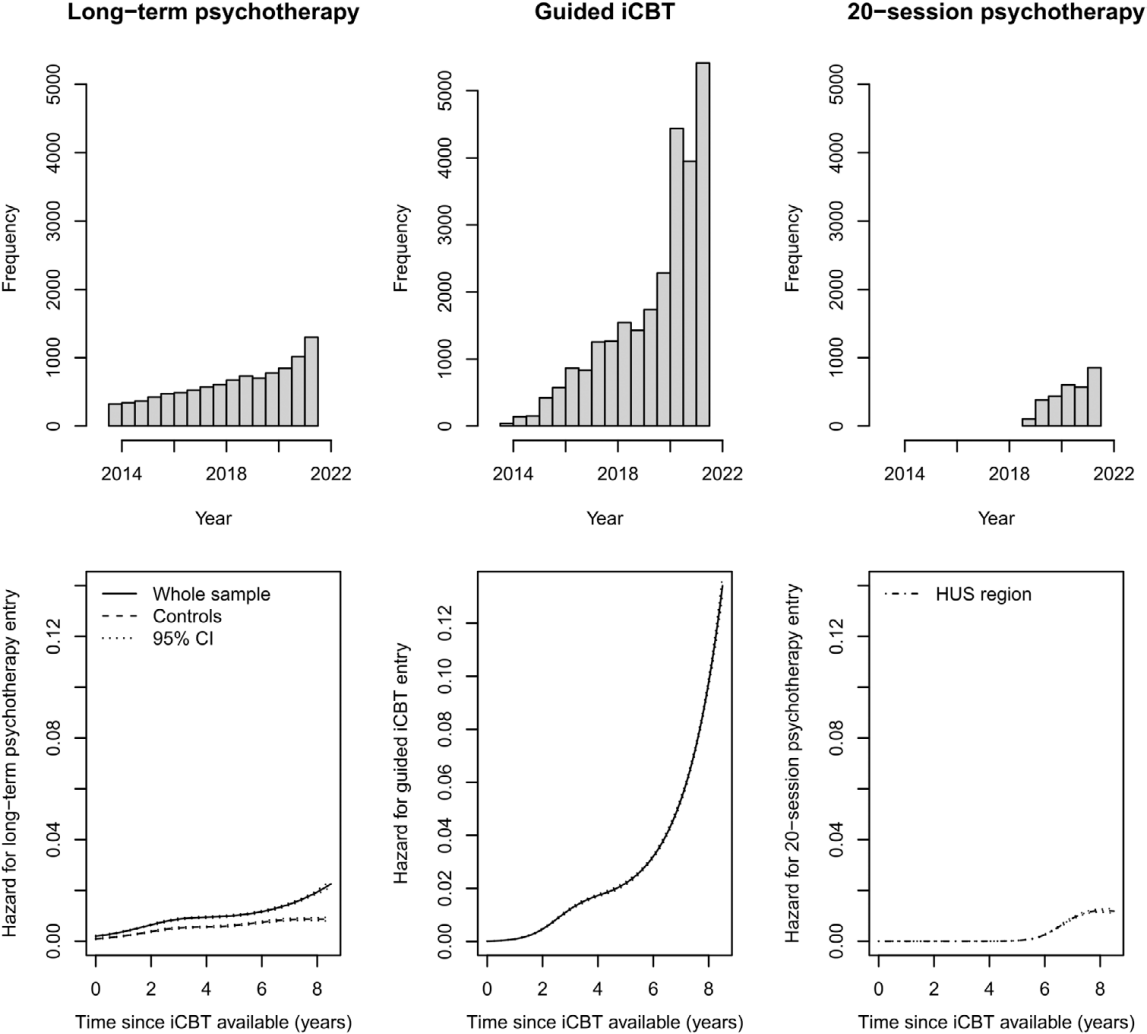
Treatment‐onset time distributions (upper row of tables) and model‐predicted onset hazard (penalized restricted cubic splines) with 95% confidence intervals (lower row) for long‐term psychotherapy, guided iCBT and ≤20‐session psychotherapy during the period August 2013 to June 2021. The tables illustrate the properties of the sample, including the different roll‐out and expansion rates of different treatments and registers relative to the time when the first HUS‐iCBT treatments became available.

The incidence of guided iCBT, long‐term and ≤20‐session psychotherapy increased over the study period (Figure 3). Consequently, the baseline hazard of initiating each treatment also increased during this time. The baseline hazard of initiating long‐term psychotherapy increased, irrespective of whether the analysis included the entire sample or was restricted to the controls.

### Proportional hazard assumptions

The proportional hazard assumption for entry into long‐term psychotherapy was not violated for guided iCBT (*χ* ^2^ = 2.85, df = 1, *p* = .091) or for ≤20‐session psychotherapy (*χ* ^2^ = 1.95, df = 1, *p* = .163). The same was true when entry into guided iCBT and ≤ 20‐session psychotherapy were used as an outcome (Table S3). Thus, the association between prior psychological treatment and subsequent relative entry rate remained constant throughout the follow‐up period, with no need for time‐dependent adjustments to balance changing proportional hazards (see Figures S1–S3 for plotted estimated effects over time). As expected, given that the proportional hazard assumption held, supplementary sensitivity analyses confirmed that the COVID‐19 pandemic did not alter these associations (see Section S5 and Table S4).

### Long‐term psychotherapy entry as an outcome

During the analysed follow‐up period, which encompassed 322,011 person‐years, altogether 5151 individuals in our sample started long‐term psychotherapy. The hazard of initiating long‐term psychotherapy was fourfold higher after therapist‐guided iCBT (HR = 4.08; CI 3.81–4.37), and nearly ninefold higher after ≤20‐session psychotherapy (HR = 8.94; CI 7.79–10.26), compared to those without these treatments, when adjusting for each other, demographics and clinical variables (Table 3; for additional models and crude HRs, see Table S5). In comparison, the first purchase of psychotropic drugs was associated with an HR of 3.85 (CI 3.56–4.16). For individuals who had had both prior psychological treatments, the hazard of initiating long‐term psychotherapy was 5.11‐fold compared to individuals with neither treatment (Table 3). Thus, the initial treatment alone increased the HR for long‐term psychotherapy, while receiving additional prior treatment did not further increase it. Comparably, when we analysed the interaction between prior psychological treatment and the first purchase of psychotropic drugs, receiving a second treatment did not increase the HR for long‐term psychotherapy to the same extent as expected from an independent effect (Table S6).

**TABLE 3:** Cox proportional hazards regression analysis for entry into long‐term psychotherapy, therapist‐guided iCBT and ≤20‐session psychotherapy; for additional models see Tables S4–S15.

Covariate	Long‐term psychotherapy		Guided iCBT		≤20‐session psychotherapy	
	HR (95% CI)^a^	*p*	HR (95% CI)^b^	*p*	HR (95% CI)^c^	*p*
Guided iCBT^d^	4.08 (3.81–4.37)	<.001	–	–	.41 (.35–.47)	<.001
≤20‐session psychotherapy^d^	8.94 (7.79–10.26)	<.001	.12 (.09–.16)	<.001	–	–
Long‐term psychotherapy^d^	–	–	.96 (.92–1.00)	.087	1.08 (.93–1.25)	.332
Interaction of prior psychological treatments^d^, ^e^	.14 (.09–.22)	<.001	.97 (.45–2.07)	.934	.53 (.34–.81)	<.05
Age^f^	.97 (.97–.97)	<.001	.99 (.99–.99)	<.001	1.02 (1.02–1.02)	<.001
Male (ref: female)	.48 (.45–.52)	<.001	1.11 (1.07–1.14)	<.001	.67 (.61–.74)	<.001
First purchase of psychotropic drugs^d^	3.85 (3.56–4.16)	<.001	6.00 (5.76–6.24)	<.001	.93 (.85–1.02)	.137
First psychiatric diagnosis^d^, ^g^	1.28 (1.19–1.38)	<.001	2.47 (2.39–2.55)	<.001	6.63 (6.05–7.27)	<.001
First purchase of paracetamol^d^	.97 (.91–1.03)	.266	.99 (.96–1.02)	.538	.62 (.56–.68)	<.001

Abbreviations: CI, confidence interval; HR, hazard ratio.

^a^ Follow‐up 322,011 person‐years from August 2018 to June 2021, during which all psychological treatments were available.

^b^ Follow‐up 311,543 person‐years from August 2018 to June 2021, during which all psychological treatments were available.

^c^ Follow‐up 132,490 person‐years from August 2018 to June 2021, during which all psychological treatments were available.

^d^ Time‐dependent covariate.

^e^ Guided iCBT x ≤20‐session psychotherapy, long‐term x ≤ 20‐session psychotherapy or guided iCBT x long‐term psychotherapy, respectively.

^f^ Patient's age at first HUS‐iCBT/FPQR record; for controls, age at index patient's first record.

^g^ First psychiatric diagnosis set in public speciality care before any treatment studied.

We estimated whether the entry rate into long‐term psychotherapy was influenced by prior treatment effectiveness. The hazard of initiating subsequent long‐term psychotherapy after guided iCBT (adjusted HR = 3.75; CI 3.51–4.01; *p* < .001) was independent of symptom change seen in the iCBT (interaction term HR = 1.02; CI .98–1.07; *p* = .322; see Table S7). The hazard of initiating long‐term psychotherapy after ≤20‐session psychotherapy (HR = 5.50; CI 4.83–6.26; *p* < .001) remained elevated, but was attenuated among patients with larger‐than‐average symptom reduction during the ≤20‐session psychotherapy, and elevated if symptom reduction was lower than average (interaction term HR = .81; CI .71–.92; *p* < .01; see Table S7). For example, a symptom reduction that was two standard deviations smaller (i.e. worse) than the average treatment effect resulted in an HR of 8.38 for entering subsequent long‐term psychotherapy, compared to an HR of 5.50 at average symptom reduction and to an HR of 3.61 among those with symptom reduction two standard deviations greater than average.

In a further sensitivity analysis using multiple imputation to account for missing iCBT symptom change data, the interaction between guided iCBT and its symptom change suggests that, under the hypothetical scenario where all patients adhered to treatment until completion, the hazard of initiating long‐term psychotherapy after iCBT was slightly attenuated among patients with larger‐than‐average symptom reduction during the guided iCBT (interaction term HR = .91; CI .86–.96; *p* < .001; Table S8). Imputation of missing ≤20‐session psychotherapy symptom change did not affect the results (Table S8).

### Guided iCBT entry as an outcome

During the analysed follow‐up period, which encompassed 311,543 person‐years, 18,532 individuals in our sample started guided iCBT. When long‐term and ≤20‐session psychotherapies were adjusted for each other, demographics and clinical variables, the hazard of subsequent iCBT entry was .12‐fold (CI .09–.16) after prior ≤20‐session psychotherapy, while prior long‐term psychotherapy was not associated with the hazard (HR = .96; CI .92–1.00; *p* = .087) (Table 3; for additional models and crude hazard ratios, see Table S9). Receiving both ≤20‐session and long‐term psychotherapy did not alter the hazard relative to the individual treatments (interaction term HR = .97; CI .45–2.07; *p* = .934) (Table 3).

Larger‐than‐average symptom reduction during ≤20‐session psychotherapy further decreased the already low hazard of entering subsequent guided iCBT (for long‐term psychotherapy, no symptom change data were available). In sensitivity analysis, the adjusted main effect was HR = .11 (CI .08–.15; *p* < .001), with an interaction term of HR = .65 (CI .47–.89; *p* < .01; see Table S10). Specifically, patients with poorer treatment outcomes (symptom reduction two standard deviations below the average treatment effect) had an HR of .26 for entering subsequent guided iCBT, compared to an HR of .11 at average symptom reduction and an HR of .05 among those with much better outcomes (two standard deviations above average). Imputation of the missing symptom change data in ≤20‐session psychotherapy did not have an effect on the results (see Table S11).

### ≤20‐session psychotherapy entry as an outcome

During the analysed follow‐up period, which encompassed 132,490 person‐years, 2476 individuals in our sample started ≤20‐session psychotherapy. The hazard of subsequent ≤20‐session psychotherapy entry was .41‐fold (CI .35–.47) after prior guided iCBT, while prior long‐term psychotherapy was not associated with the hazard (HR = 1.08; CI .93–1.25; *p* = .332) when the therapies were adjusted for each other, demographics and clinical variables (Table 3; for additional models and crude hazard ratios, see Table S12). However, receiving both prior guided iCBT and long‐term psychotherapy was associated with an even lower hazard of starting ≤20‐session psychotherapy than receiving iCBT alone (Table 3). In contrast to other outcomes, the first purchase of psychotropic drugs was associated with a lower hazard of subsequently initiating ≤20‐session psychotherapy. Similarly, the negative control, first purchase of a paracetamol prescription, was also associated with a reduced hazard of entering ≤20‐session psychotherapy; however, unlike psychotropics, this association was explained by its interaction effect with age (Table S13).

Larger‐than‐average symptom reduction during guided iCBT was associated with a further decrease in the hazard of entering subsequent ≤20‐session psychotherapy: the adjusted main effect was HR = .33 (CI .28–.38; *p* < .001), with an interaction term of HR = .51 (CI .44–.59; *p* < .001; see Table S14). For long‐term psychotherapy, no symptom change data were available. Specifically, patients with poorer treatment outcomes during guided iCBT (symptom reduction two standard deviations below the average treatment effect) had an HR of 1.27 for entering subsequent ≤20‐session psychotherapy, compared to an HR of .33 at average symptom reduction and an HR of .09 among those with much better outcomes (two standard deviations above average). Imputation of the missing iCBT symptom change data did not have an effect on the results (see Table S15).

## DISCUSSION

This register study included 126,719 individuals followed for several years within Finnish routine care. We found that initial therapist‐guided iCBT or ≤20‐session psychotherapy was associated with increased entry rates for subsequent long‐term psychotherapy, but reduced entry rates for each other. The entry rate for long‐term psychotherapy was four times higher after iCBT and nearly nine times higher after ≤20‐session psychotherapy, compared to those without these treatments (adjusted for each other, age, sex and clinical variables). However, the majority—approximately four out of five patients—in the guided iCBT and ≤20‐session psychotherapy groups did not receive either prior or subsequent long‐term psychotherapy by the end of follow‐up. Notably, symptom reduction during guided iCBT did not influence the entry rate to subsequent long‐term face‐to‐face psychotherapy, whereas it moderated the likelihood of entering shorter, ≤20‐session face‐to‐face psychotherapy. In contrast, symptom reduction in ≤20‐session psychotherapy moderated the magnitude, but not the direction, of the therapy's effect on entry rate to subsequent therapy.

Despite increased treatment volumes and the COVID‐19 pandemic, these relative effects of prior psychological treatment on entering subsequent treatment remained stable during follow‐up for all psychological treatments investigated. Thus, we did not find empirical evidence that receiving multiple subsequent therapies had accelerated during the follow‐up, despite recent media coverage speculating about the negative consequences of increased mental health awareness (Foulkes, 2024).

Initial treatment can influence subsequent treatments in several ways (e.g. Kilcullen et al., 2021). Adequate treatment may reduce the need for further interventions, while inadequate treatment or adverse effects may require additional care. Negative experiences can decrease motivation, whereas positive experiences can increase willingness to seek further treatment. We found that guided iCBT and ≤ 20‐session psychotherapy were linked to a higher entry rate to subsequent long‐term psychotherapy, despite their demonstrated effectiveness in reducing symptoms in routine care (Ritola et al., 2022; Rosenström et al., 2025; Saarni et al., 2023; Stenberg et al., 2022). In Finland, at least 3 months of appropriate treatment is a statutory requirement for access to long‐term rehabilitative psychotherapy, but not for iCBT or ≤ 20‐session psychotherapy. This is a plausible explanation for why prior psychological treatment was associated with an increased entry rate for long‐term psychotherapy but not for guided iCBT or ≤ 20‐session psychotherapy. If the original treatment plan aims for long‐term psychotherapy with short‐term treatment as a gateway to extended care, previous treatment may lead to long‐term therapy regardless of the outcome. Indeed, we found that after guided iCBT, the entry rate for subsequent face‐to‐face psychotherapy was independent of symptom change during iCBT only for long‐term psychotherapy, not for shorter, ≤20‐session psychotherapy. In addition, even patients with much better than average symptom improvement during ≤20‐session psychotherapy still had a substantially elevated entry rate for long‐term psychotherapy, comparable to that observed after guided iCBT. Furthermore, although prior treatment was associated with a higher entry rate for long‐term psychotherapy, additional prior treatment did not further increase it. The lack of dose–response relationship also supports the gateway theory.

However, since it remains unclear whether people entered long‐term psychotherapy for the same reasons as their initial treatment, we cannot rule out that shorter‐term interventions increased the entry rate for subsequent treatment because they indicated a need but were inadequate responses to it. In the UK, 58% of patients who returned to mental health services had had enduring, clinically significant symptoms at the end of their first psychological treatment, yet the effect size indicated that recovery was only negligibly associated with treatment return (Lorimer et al., 2024). Broadly aligning, we found that entry rate to subsequent long‐term face‐to‐face psychotherapy was independent of symptom reduction during guided iCBT. Although symptom change during ≤20‐session psychotherapy significantly moderated the hazard of subsequent long‐term psychotherapy, the hazard ratio remained elevated even for the patients with larger‐than‐average symptom reduction. Thus, treatment's success at post‐treatment slightly moderated, but did not strongly determine, the effect of ≤20‐session psychotherapy on entry into subsequent treatments. In contrast, the treatment effect of guided iCBT was pronounced for shorter, ≤20‐session face‐to‐face psychotherapy, where very poor outcomes even reversed the direction of likelihood for subsequent face‐to‐face psychotherapy. Altogether, these findings suggest that receiving subsequent long‐term psychotherapy, particularly after guided iCBT, stemmed from factors independent of the reduction seen in the target disorder's symptoms, further supporting the gateway theory.

As our analysis focused on immediate pre‐post symptom change and did not account for baseline severity or maintenance of gains over time, residual symptoms or relapse could still partly explain the lack of systematic association between symptom change and entry rates to subsequent treatments. It is possible that some individuals who initially benefited from treatment later experienced a return of symptoms, prompting further treatment. In the United Kingdom, 32% of returning patients had had a relapse by the beginning of the second treatment (Lorimer et al., 2024). Altogether, while residual symptoms are known to predict relapse (e.g. Buckman et al., 2018; Delgadillo et al., 2018; Palacios et al., 2022; Wojnarowski et al., 2019), actual utilization of mental health services is shaped by various individual and contextual factors (Roberts et al., 2018). Future research is needed to clarify whether broader or transdiagnostic measurements of psychological distress would have additional value in predicting future treatment use, given the high comorbidity and cumulative risk associated with mental disorders (McGrath et al., 2020; Menzies et al., 2024). However, findings from studies in Spain and Norway imply that symptom reduction plays an important role in explaining functional improvements (Smith et al., 2023), as well as in other outcomes of societal and economic relevance, such as reduced absenteeism and health care costs (Barrio‐Martínez et al., 2024; Smith et al., 2025).

The magnitude difference in entry rates for long‐term psychotherapy after ≤20‐session psychotherapy versus iCBT was unexpected, given that therapist‐guided iCBT frequently yields similar symptom outcomes as face‐to‐face therapy (Andersson et al., 2018; Cuijpers et al., 2010; Mamukashvili‐Delau et al., 2023), as has also been shown in a Finnish sample from partly the same registers used in the current study (Rosenström et al., 2025). We found that the entry rate for long‐term psychotherapy was roughly double after face‐to‐face ≤20‐session psychotherapy compared to therapist‐guided iCBT or the first purchase of psychotropic drugs. Additionally, prior therapist‐guided iCBT was associated with a lower entry rate for ≤20‐session psychotherapy, while prior face‐to‐face long‐term psychotherapy was not. One possible explanation for these findings is that digital and pharmacological treatments reach patients who would not choose face‐to‐face psychotherapy (e.g. 12.6% in a South African student sample, see Gbollie et al., 2023). Conversely, some individuals prefer face‐to‐face psychotherapy and may be referred to it based on professional recommendations, particularly when this more intensive care is deemed necessary. Becoming familiar with it may increase their readiness to receive similar help, if found useful. Practically, it may also be easier to access long‐term psychotherapy if one has a familiar therapist to contact, as it is possible to continue therapy with the same provider.

Therapist‐guided iCBT and ≤ 20‐session psychotherapy treatments were associated with lower entry rates for each other, whereas prior long‐term psychotherapy showed no independent association with subsequent shorter‐term treatment. Thus, longer treatments were not consistently associated with reduced subsequent treatment use, broadly aligning with previous research reporting weak or negligible associations between treatment duration and return to treatment (Boerema et al., 2016; Lorimer et al., 2024).

Altogether, our results suggest that patients receiving therapist‐guided iCBT are in general less likely to access subsequent face‐to‐face psychotherapy than those whose initial treatment mode was face‐to‐face in Finnish routine care.

### Limitations

Our findings should be interpreted considering certain limitations. Although considered as a sign of causation, temporal precedence does not prove causation (Hill, 1965). The sample was not a population‐based random sample but derived from comprehensive registers of patients receiving certain treatments and their population controls. Thus, the exact hazard estimates are not generalizable for long‐term psychotherapy at the population level, although we expect similar hazard ratios and temporal precedence. Psychiatric diagnoses were only from public specialty care, excluding primary care and the private sector, due to differences in data registration practices and reporting obligations to national registries. This might explain why around 30% of patients had no recorded diagnosis. We also lacked complete information on all treatments received outside public health care and could not determine if patients continued with the same therapist from ≤20‐session psychotherapy to long‐term psychotherapy. Finally, we were not able to estimate the effects of symptom change during long‐term psychotherapy, as the data were not available.

### Strengths

The study's strengths include extensive long‐term follow‐up data from a naturalistic setting including a comprehensive sample of patients receiving two different psychological treatments within public health care, providing good external validity among people with mental disorders and addressing common methodological issues in long‐term treatment studies. The treatments studied varied in intensity, and to the best of our knowledge, this is the first large‐scale study estimating the return to treatment following iCBT. All participants, whether receiving interventions or not, were equally followed up, with complete coverage of intervention entries for all treatments studied and no attrition between the end of treatment and follow‐up. Long naturalistic follow‐ups allow for examining changes over time and assessing the temporality and reversibility between a presumed cause and an observed effect, which are relevant for causal inference (Hill, 1965). Unlike previous naturalistic studies of return to treatment, which were limited to single‐service data (Boerema et al., 2016; Lorimer et al., 2024), this study captured all transitions from treatments provided by Finland's largest hospital to nationwide rehabilitative long‐term psychotherapy.

## CONCLUSION

While deepening our understanding of treatment sufficiency and return to care is crucial, systemic factors within mental health services complicate conclusions about psychological treatment adequacy. This was underscored by our findings from Finland, where prior treatment is a prerequisite for accessing long‐term psychotherapy. Therapist‐guided iCBT and ≤ 20‐session psychotherapy were associated with reduced entry rates into each other, but with increased entry rates into very long‐term psychotherapy—particularly among face‐to‐face psychotherapy recipients. The predictive value of treatment outcome for subsequent treatment entry was neither consistent nor uniform.

Our findings highlight the importance of critically evaluating treatment contingencies and whether they are driven by patient needs or shaped by system‐level constraints. Our results suggest that besides treatment effectiveness, contingencies between treatment services should be monitored and factored into service‐system development and its cost‐effectiveness considerations. Future research should continue to disentangle clinical need from service systems effects to better understand treatment continuation patterns.

## AUTHOR CONTRIBUTIONS

**A. Plattonen:** Conceptualization; formal analysis; writing – original draft; writing – review and editing. **S. Mylläri:** Writing – review and editing; supervision. **S. E. Saarni:** Conceptualization; writing – review and editing; supervision; funding acquisition. **T. Rosenström:** Conceptualization; methodology; writing – review and editing; supervision; funding acquisition.

## FUNDING INFORMATION

This work was supported by the Department of Psychiatry, Helsinki University Hospital (AP), the Academy of Finland (TR, grant numbers 334057, 335901, 358138), the Social Insurance Institution of Finland (AP, SES, Kela 140/331/2021), the Medical Society of Finland (SES, Finska Läkaresällskapet rf) and the European Union—NextGenerationEU (AP, SES). The funders had no role in the study design, data collection and analysis, decision to publish or preparation of the manuscript.

## CONFLICT OF INTEREST STATEMENT

AP and SES are employed by Helsinki University Hospital (HUS), Division of Digital and Psychosocial Treatments (listed as an affiliation). HUS is a publicly funded hospital providing national iCBT services, short face‐to‐face psychotherapies and therapist training. Neither AP nor SES has received personal financial compensation from these activities. Both are small‐scale private providers of one of the treatments studied (long‐term rehabilitative psychotherapy supported by the Social Insurance Institution of Finland). SES has also served in a non‐remunerated fiduciary role in the national guideline working group for obsessive‐compulsive disorder (OCD). Authors have academic roles (research and teaching) related to therapeutic interventions and have received compensation for lectures or presentations on this topic. Apart from these, the authors have no further conflicts of interest to disclose.

## ETHICS STATEMENT

Our research permission was granted by the Finnish Social and Health Data Permit Authority Findata (THL/4810/14.02.00/2020 and THL/1303/14.06.00/2023). As the study used only register data, no informed consent was required. The study was approved by the Ethical Committee of HUS (HUS/3150/2020).

## Supporting information


Data S1:

## Data Availability

Access to data is available via the Finnish Social and Health Data Permit Authority Findata by applying for a permit for the secondary use of social and health care registers.

## References

[bjc70036-bib-0001] Andersson, G. , Hesser, H. , Hummerdal, D. , Bergman‐Nordgren, L. , & Carlbring, P. (2013). A 3.5‐year follow‐up of internet‐delivered cognitive behavior therapy for major depression. Journal of Mental Health, 22(2), 155–164. 10.3109/09638237.2011.608747 21957933

[bjc70036-bib-0002] Andersson, G. , Rozental, A. , Shafran, R. , & Carlbring, P. (2018). Long‐term effects of internet‐supported cognitive behaviour therapy. Expert Review of Neurotherapeutics, 18(1), 21–28. 10.1080/14737175.2018.1400381 29094622

[bjc70036-bib-0003] Baigent, M. , Smith, D. , Battersby, M. , Lawn, S. , Redpath, P. , & McCoy, A. (2023). The Australian version of IAPT: Clinical outcomes of the multi‐site cohort study of NewAccess. Journal of Mental Health, 32(1), 341–350. 10.1080/09638237.2020.1760224 32394756

[bjc70036-bib-0004] Barrio‐Martínez, S. , Ruiz‐Rodríguez, P. , Medrano, L. A. , Priede, A. , Muñoz‐Navarro, R. , Moriana, J. A. , Carpallo‐González, M. , Prieto‐Vila, M. , Cano‐Vindel, A. , & González‐Blanch, C. (2024). Effect of reliable recovery on health care costs and productivity losses in emotional disorders. Behavior Therapy, 55(3), 585–594. 10.1016/j.beth.2023.08.012 38670670

[bjc70036-bib-0005] Boerema, A. M. , Cuijpers, P. , Beekman, A. T. F. , Hellenthal, A. , Voorrips, L. , & van Straten, A. (2016). Is duration of psychological treatment for depression related to return into treatment? Social Psychiatry and Psychiatric Epidemiology, 51(11), 1495–1507. 10.1007/s00127-016-1267-7 27448572 PMC5101270

[bjc70036-bib-0006] Bruin, E. K. , Scholten, W. , Muntingh, A. , Maarsingh, O. , van Meijel, B. , van Straten, A. , & Batelaan, N. (2022). Psychological interventions to prevent relapse in anxiety and depression: A systematic review and meta‐analysis. PLoS One, 17(8), e0272200. 10.1371/journal.pone.0272200 35960783 PMC9374222

[bjc70036-bib-0007] Buckman, J. E. J. , Underwood, A. , Clarke, K. , Saunders, R. , Hollon, S. D. , Fearon, P. , & Pilling, S. (2018). Risk factors for relapse and recurrence of depression in adults and how they operate: A four‐phase systematic review and meta‐synthesis. Clinical Psychology Review, 64, 13–38. 10.1016/j.cpr.2018.07.005 30075313 PMC6237833

[bjc70036-bib-0008] van Buuren, S. , & Groothuis‐Oudshoorn, K. (2011). Mice: Multivariate imputation by chained equations in R. Journal of Statistical Software, 45, 1–67. 10.18637/jss.v045.i03

[bjc70036-bib-0009] Cox, D. R. (1972). Regression models and life‐tables. Journal of the Royal Statistical Society. Series B, Statistical Methodology, 34(2), 187–202. 10.1111/j.2517-6161.1972.tb00899.x

[bjc70036-bib-0010] Cuijpers, P. , Donker, T. , van Straten, A. , Li, J. , & Andersson, G. (2010). Is guided self‐help as effective as face‐to‐face psychotherapy for depression and anxiety disorders? A systematic review and meta‐analysis of comparative outcome studies. Psychological Medicine, 40(12), 1943–1957. 10.1017/S0033291710000772 20406528

[bjc70036-bib-0011] Cuijpers, P. , Miguel, C. , Ciharova, M. , Harrer, M. , Basic, D. , Cristea, I. A. , de Ponti, N. , Driessen, E. , Hamblen, J. , Larsen, S. E. , Matbouriahi, M. , Papola, D. , Pauley, D. , Plessen, C. Y. , Pfund, R. A. , Setkowski, K. , Schnurr, P. P. , van Ballegooijen, W. , Wang, Y. , & Karyotaki, E. (2024). Absolute and relative outcomes of psychotherapies for eight mental disorders: A systematic review and meta‐analysis. World Psychiatry, 23(2), 267–275. 10.1002/wps.21203 PMC11083862 38727072

[bjc70036-bib-0012] Cuijpers, P. , Miguel, C. , Harrer, M. , Plessen, C. Y. , Ciharova, M. , Ebert, D. , & Karyotaki, E. (2023). Cognitive behavior therapy vs. control conditions, other psychotherapies, pharmacotherapies and combined treatment for depression: A comprehensive meta‐analysis including 409 trials with 52,702 patients. World Psychiatry, 22(1), 105–115. 10.1002/wps.21069 PMC9840507 36640411

[bjc70036-bib-0013] Delgadillo, J. , Ali, S. , Fleck, K. , Agnew, C. , Southgate, A. , Parkhouse, L. , Cohen, Z. D. , DeRubeis, R. J. , & Barkham, M. (2022). Stratified care vs stepped Care for Depression: A cluster randomized clinical trial. JAMA Psychiatry, 79(2), 101–108. 10.1001/jamapsychiatry.2021.3539 PMC8655665 34878526

[bjc70036-bib-0014] Delgadillo, J. , Rhodes, L. , Moreea, O. , McMillan, D. , Gilbody, S. , Leach, C. , Lucock, M. , Lutz, W. , & Ali, S. (2018). Relapse and recurrence of common mental health problems after low intensity cognitive Behavioural therapy: the WYLOW longitudinal cohort study. Psychotherapy and Psychosomatics, 87(2), 116–117. 10.1159/000485386 29462816

[bjc70036-bib-0015] Foulkes, L. (2024). The adolescent mental health mess. Medium. https://lucyfoulkes3.medium.com/the‐adolescent‐mental‐health‐mess‐c93f23f8ed56

[bjc70036-bib-0016] Furukawa, T. A. , Shinohara, K. , Sahker, E. , Karyotaki, E. , Miguel, C. , Ciharova, M. , Bockting, C. L. H. , Breedvelt, J. J. F. , Tajika, A. , Imai, H. , Ostinelli, E. G. , Sakata, M. , Toyomoto, R. , Kishimoto, S. , Ito, M. , Furukawa, Y. , Cipriani, A. , Hollon, S. D. , & Cuijpers, P. (2021). Initial treatment choices to achieve sustained response in major depression: A systematic review and network meta‐analysis. World Psychiatry, 20(3), 387–396. 10.1002/wps.20906 34505365 PMC8429344

[bjc70036-bib-0017] Gbollie, E. F. , Bantjes, J. , Jarvis, L. , Swandevelder, S. , du Plessis, J. , Shadwell, R. , Davids, C. , Gerber, R. , Holland, N. , & Hunt, X. (2023). Intention to use digital mental health solutions: A cross‐sectional survey of university students attitudes and perceptions toward online therapy, mental health apps, and chatbots. Digital Health, 9, 20552076231216559. 10.1177/20552076231216559 38047161 PMC10693229

[bjc70036-bib-0018] Grambsch, P. M. , & Therneau, T. M. (1994). Proportional hazards tests and diagnostics based on weighted residuals. Biometrika, 81(3), 515–526. 10.1093/biomet/81.3.515

[bjc70036-bib-0019] Hedman‐Lagerlöf, E. , Carlbring, P. , Svärdman, F. , Riper, H. , Cuijpers, P. , & Andersson, G. (2023). Therapist‐supported internet‐based cognitive behaviour therapy yields similar effects as face‐to‐face therapy for psychiatric and somatic disorders: An updated systematic review and meta‐analysis. World Psychiatry, 22(2), 305–314. 10.1002/wps.21088 PMC10168168 37159350

[bjc70036-bib-0020] Hill, A. B. (1965). The environment and disease: Association or causation? Proceedings of the Royal Society of Medicine, 58(5), 295–300. 10.1177/003591576505800503 PMC1898525 14283879

[bjc70036-bib-0021] Kilcullen, R. J. , Castonguay, L. G. , Janis, R. A. , Hallquist, M. N. , Hayes, J. A. , & Locke, B. D. (2021). Predicting future courses of psychotherapy within a grouped LASSO framework. Psychotherapy Research, 31(1), 63–77. 10.1080/10503307.2020.1762948 32406339

[bjc70036-bib-0022] Knapstad, M. , Nordgreen, T. , & Smith, O. R. F. (2018). Prompt mental health care, the Norwegian version of IAPT: Clinical outcomes and predictors of change in a multicenter cohort study. BMC Psychiatry, 18(1), 260. 10.1186/s12888-018-1838-0 PMC6097447 30115041

[bjc70036-bib-0023] Knekt, P. , Lindfors, O. , Renlund, C. , Sares‐Jäske, L. , Laaksonen, M. A. , & Virtala, E. (2011). Use of auxiliary psychiatric treatment during a 5‐year follow‐up among patients receiving short‐ or long‐term psychotherapy. Journal of Affective Disorders, 135(1–3), 221–230. 10.1016/j.jad.2011.07.024 21871667

[bjc70036-bib-0024] Kobori, O. , Nakazato, M. , Yoshinaga, N. , Shiraishi, T. , Takaoka, K. , Nakagawa, A. , Iyo, M. , & Shimizu, E. (2014). Transporting cognitive behavioral therapy (CBT) and the improving access to psychological therapies (IAPT) project to Japan: Preliminary observations and Service evaluation in Chiba. The Journal of Mental Health Training, Education and Practice, 9, 155–166. 10.1108/JMHTEP-10-2013-0033

[bjc70036-bib-0025] Levy, H. C. , O'Bryan, E. M. , & Tolin, D. F. (2021). A meta‐analysis of relapse rates in cognitive‐behavioral therapy for anxiety disorders. Journal of Anxiety Disorders, 81, 102407. 10.1016/j.janxdis.2021.102407 33915506

[bjc70036-bib-0026] Lorimer, B. , Kellett, S. , Giesemann, J. , Lutz, W. , & Delgadillo, J. (2024). An investigation of treatment return after psychological therapy for depression and anxiety. Behavioural and Cognitive Psychotherapy, 52(2), 149–162. 10.1017/S1352465823000322 37563726

[bjc70036-bib-0027] Maljanen, T. , Knekt, P. , Lindfors, O. , Virtala, E. , Tillman, P. , Härkänen, T. , & Helsinki Psychotherapy Study Group . (2016). The cost‐effectiveness of short‐term and long‐term psychotherapy in the treatment of depressive and anxiety disorders during a 5‐year follow‐up. Journal of Affective Disorders, 190, 254–263. 10.1016/j.jad.2015.09.065 26540079

[bjc70036-bib-0028] Mamukashvili‐Delau, M. , Koburger, N. , Dietrich, S. , & Rummel‐Kluge, C. (2023). Long‐term efficacy of internet‐based cognitive behavioral therapy self‐help programs for adults with depression: Systematic review and meta‐analysis of randomized controlled trials. JMIR Mental Health, 10(1), e46925. 10.2196/46925 PMC10481211 37606990

[bjc70036-bib-0029] McGrath, J. J. , Lim, C. C. W. , Plana‐Ripoll, O. , Holtz, Y. , Agerbo, E. , Momen, N. C. , Mortensen, P. B. , Pedersen, C. B. , Abdulmalik, J. , Aguilar‐Gaxiola, S. , Al‐Hamzawi, A. , Alonso, J. , Bromet, E. J. , Bruffaerts, R. , Bunting, B. , de Almeida, J. M. C. , de Girolamo, G. , Vries, Y. A. D. , Florescu, S. , & de Jonge, P. (2020). Comorbidity within mental disorders: A comprehensive analysis based on 145 990 survey respondents from 27 countries. Epidemiology and Psychiatric Sciences, 29, e153. 10.1017/S2045796020000633 PMC7443806 32782057

[bjc70036-bib-0030] Menzies, R. E. , Richmond, B. , Sharpe, L. , Skeggs, A. , Liu, J. , & Coutts‐Bain, D. (2024). The ‘revolving door’ of mental illness: A meta‐analysis and systematic review of current versus lifetime rates of psychological disorders. British Journal of Clinical Psychology, 63(2), 178–196. 10.1111/bjc.12453 38197576

[bjc70036-bib-0031] Ormel, J. , Hollon, S. D. , Kessler, R. C. , Cuijpers, P. , & Monroe, S. M. (2022). More treatment but no less depression: The treatment‐prevalence paradox. Clinical Psychology Review, 91, 102111. 10.1016/j.cpr.2021.102111 34959153

[bjc70036-bib-0032] Palacios, J. E. , Enrique, A. , Mooney, O. , Farrell, S. , Earley, C. , Duffy, D. , Eilert, N. , Harty, S. , Timulak, L. , & Richards, D. (2022). Durability of treatment effects following internet‐delivered cognitive behavioural therapy for depression and anxiety delivered within a routine care setting. Clinical Psychology & Psychotherapy, 29(5), 1768–1777. 10.1002/cpp.2743 PMC9790710 35466486

[bjc70036-bib-0033] Reeder, K. , Park, A. L. , & Chorpita, B. F. (2020). Turning Back to treatment: The effect of attendance and symptom outcomes on subsequent Service use. Administration and Policy in Mental Health and Mental Health Services Research, 47(4), 641–647. 10.1007/s10488-020-01032-3 32170492

[bjc70036-bib-0034] Ritola, V. , Lipsanen, J. O. , Pihlaja, S. , Gummerus, E.‐M. , Stenberg, J.‐H. , Saarni, S. , & Joffe, G. (2022). Internet‐delivered cognitive behavioral therapy for generalized anxiety disorder in Nationwide routine care: Effectiveness study. Journal of Medical Internet Research, 24(3), e29384. 10.2196/29384 PMC8990365 35323119

[bjc70036-bib-0035] Roberts, T. , Esponda, G. M. , Krupchanka, D. , Shidhaye, R. , Patel, V. , & Rathod, S. (2018). Factors associated with health service utilisation for common mental disorders: A systematic review. BMC Psychiatry, 18, 262. 10.1186/s12888-018-1837-1 30134869 PMC6104009

[bjc70036-bib-0036] Rosenström, T. H. , Ritola, V. , Saarni, S. , Joffe, G. , & Stenberg, J.‐H. (2023). Measurement invariant but non‐normal treatment responses in guided internet psychotherapies for depressive and generalized anxiety disorders. Assessment, 30(3), 618–632. 10.1177/10731911211062500 PMC9999284 34905968

[bjc70036-bib-0037] Rosenström, T. H. , Saarni, S. E. , Saarni, S. I. , Tammilehto, J. , & Stenberg, J.‐H. (2025). Efficacy and effectiveness of therapist‐guided internet versus face‐to‐face cognitive behavioural therapy for depression via counterfactual inference using naturalistic registers and machine learning in Finland: A retrospective cohort study. The Lancet Psychiatry, 12(3), 189–197. 10.1016/S2215-0366(24)00404-8 39954684

[bjc70036-bib-0038] Saarni, S. E. , Rosenström, T. , Stenberg, J.‐H. , Plattonen, A. , Holi, M. , Ekelund, J. , Granö, N. , Komsi, N. , & Saarni, S. I. (2023). Finnish psychotherapy quality register: Rationale, development, and baseline results. Nordic Journal of Psychiatry, 77(5), 455–466. 10.1080/08039488.2022.2150788 36541920

[bjc70036-bib-0039] Saarni, S. I. , Nurminen, S. , Mikkonen, K. , Service, H. , Karolaakso, T. , Stenberg, J.‐H. , Ekelund, J. , & Saarni, S. (2022). The Finnish therapy navigator – Digital support system for introducing stepped care in Finland. Psychiatria Fennica, 53, 120–137.

[bjc70036-bib-0040] Santomauro, D. F. , Vos, T. , Whiteford, H. A. , Chisholm, D. , Saxena, S. , & Ferrari, A. J. (2024). Service coverage for major depressive disorder: Estimated rates of minimally adequate treatment for 204 countries and territories in 2021. The Lancet Psychiatry, 11(12), 1012–1021. 10.1016/S2215-0366(24)00317-1 PMC11579305 39572105

[bjc70036-bib-0041] Smith, O. R. F. , Aarø, L. E. , & Knapstad, M. (2023). The importance of symptom reduction for functional improvement after cognitive behavioral therapy for anxiety and depression: A causal mediation analysis. Psychotherapy and Psychosomatics, 92(3), 193–202. 10.1159/000530650 37231987

[bjc70036-bib-0042] Smith, O. R. F. , Clark, D. M. , Hensing, G. , Layard, R. , & Knapstad, M. (2025). Cost–benefit of IAPT Norway and effects on work‐related outcomes and health care utilization: Results from a randomized controlled trial using registry‐based data. Psychological Medicine, 55, e86. 10.1017/S003329172500025X 40719346 PMC12080634

[bjc70036-bib-0043] Social Insurance Institution of Finland . (2022). Kelan kuntoutustilasto 2021. http://hdl.handle.net/10138/343057

[bjc70036-bib-0044] Stenberg, J.‐H. , Ritola, V. , Joffe, G. , Saarni, S. , & Rosenström, T. (2022). Effectiveness of mobile‐delivered, therapist‐assisted cognitive behavioral therapy for insomnia in nationwide routine clinical care in Finland. Journal of Clinical Sleep Medicine, 18, 2643–2651. 10.5664/jcsm.10186 35929590 PMC9622989

[bjc70036-bib-0045] Thornicroft, G. , Chatterji, S. , Evans‐Lacko, S. , Gruber, M. , Sampson, N. , Aguilar‐Gaxiola, S. , Al‐Hamzawi, A. , Alonso, J. , Andrade, L. , Borges, G. , Bruffaerts, R. , Bunting, B. , de Almeida, J. M. C. , Florescu, S. , de Girolamo, G. , Gureje, O. , Haro, J. M. , He, Y. , Hinkov, H. , & Kessler, R. C. (2017). Undertreatment of people with major depressive disorder in 21 countries. British Journal of Psychiatry, 210(2), 119–124. 10.1192/bjp.bp.116.188078 PMC5288082 27908899

[bjc70036-bib-0046] Vigo, D. , Haro, J. M. , Hwang, I. , Aguilar‐Gaxiola, S. , Alonso, J. , Borges, G. , Bruffaerts, R. , Caldas‐de‐Almeida, J. M. , de Girolamo, G. , Florescu, S. , Gureje, O. , Karam, E. , Karam, G. , Kovess‐Masfety, V. , Lee, S. , Navarro‐Mateu, F. , Ojagbemi, A. , Posada‐Villa, J. , Sampson, N. A. , & Kessler, R. C. (2020). Towards measuring effective treatment coverage: Critical bottlenecks in quality‐ and user‐adjusted coverage for major depressive disorder. Psychological Medicine, 52, 1–11. 10.1017/S0033291720003797 PMC9341444 33077023

[bjc70036-bib-0047] Wakefield, S. , Kellett, S. , Simmonds‐Buckley, M. , Stockton, D. , Bradbury, A. , & Delgadillo, J. (2021). Improving access to psychological therapies (IAPT) in the United Kingdom: A systematic review and meta‐analysis of 10‐years of practice‐based evidence. British Journal of Clinical Psychology, 60(1), e12259. 10.1111/bjc.12259 32578231

[bjc70036-bib-0048] Wojnarowski, C. , Firth, N. , Finegan, M. , & Delgadillo, J. (2019). Predictors of depression relapse and recurrence after cognitive behavioural therapy: A systematic review and meta‐analysis. Behavioural and Cognitive Psychotherapy, 47(5), 514–529. 10.1017/S1352465819000080 30894231

