# Supplementary material for: Therapy after therapy: Entry rates into subsequent psychological treatment among patients receiving therapist‐guided internet‐delivered or face‐to‐face psychotherapy
Source: Br J Clin Psychol. 2026 Jan 5;65(2):451–69. doi: 10.1111/bjc.70036 (PMC13159770; doi:10.1111/bjc.70036)
Supplement: Supplementary file 1 — Data S1: [file BJC-65-451-s001.docx]

Supplementary material

# Therapy after therapy: Entry rates into subsequent psychological treatment among patients receiving therapist-guided internet-delivered or face-to-face psychotherapy

Table of Contents

[1. Considerations on covariate selection 2](#_Toc215145254)

[2. Primary measures in guided iCBT programs and ≤20-session psychotherapy 3](#_Toc215145255)

[3. Testing assumption of proportional hazards 4](#_Toc215145256)

[4. Consideration of missing symptom change data 8](#_Toc215145257)

[5. COVID-19 and its impact on treatment delivery 9](#_Toc215145258)

[6. Sensitive analysis for entry into long-term psychotherapy 11](#_Toc215145259)

[7. Sensitive analysis for entry into therapist-guided iCBT 15](#_Toc215145260)

[8. Sensitive analysis for entry into ≤20-session psychotherapy 17](#_Toc215145261)

### Considerations on covariate selection

**Table S1.** Rationale for each covariate

| **Covariate** | **Rationale** |  |
| --- | --- | --- |
| Age | Patients' age may influence treatment outcomes (e.g., older patients benefit more from iCBT, while young adults may respond best to face-to-face therapy (Catarino et al., 2018; Cuijpers et al., 2020; Karyotaki et al., 2018; Reins et al., 2020)) |  |
| Sex | Gender is associated with likelihood of diagnosed mental health disorder and mental health service utilization (e.g. female gender is associated with increased lifetime incidence of mental health disorders(Kessing et al., 2023; Suokas et al., 2025), and increased likelihood of using mental health care services including rehabilitative psychotherapy in Finland (Leppänen et al., 2022; Selinheimo et al., 2023); female gender also increases adherence to internet-based therapies (Beatty and Binnion, 2016; Karyotaki et al., 2015) |  |
| First purchase of psychotropic drugs | Along with psychotherapies, psychotropic drugs are major treatment modality for common mental health disorders, and pharmacological treatments serve as usual first line treatments used before referral to rehabilitation psychotherapy in Finland |  |
| First psychiatric diagnosis | Psychotherapies are indicated for mental health disorders and psychiatric diagnosis is a service-system prerequisite for treatments studied, but due to data available indicated here using public specialty care as a service pathway |  |
| First purchase of paracetamol under prescription | Paracetamol, which has no approved indication for mental health disorders, was used as a negative control (yet physical pain is a risk factor for depression (Stubbs et al., 2017)) |  |
| Symptom change | Symptom change may moderate the effect of prior therapy on subsequent treatment: residual symptoms are linked to relapse risk (e.g. (Buckman et al., 2018; Delgadillo et al., 2018; Palacios et al., 2022; Wojnarowski et al., 2019), while better treatment outcomes are associated with e.g. functional improvements (Smith et al., 2023), reduced absenteeism and health-care costs (Barrio-Martínez et al., 2024; Smith et al., 2025); however, symptom recovery does not always correlate with treatment return (Lorimer et al., 2024), and combined with high therapy attendance may even increase return to treatment (Reeder et al., 2020). |  |
|  | | |

### Primary measures in guided iCBT programs and ≤20-session psychotherapy

In all HUS iCBT-programs patient fills validated symptom severity questionnaires according to focus of the treatment and in predefined sessions or at every session, and at three months follow-up after treatment completion. Symptom changes for all iCBT-programs are available in Finnish language (HUS, 2025). In outsourced ≤20-session psychotherapy provided by HUS, both the patient and therapist complete questionnaires for the Finnish Psychotherapy Quality Register (FPQR) at the beginning and end of treatment. These questionnaires include validated measures of symptom severity and functioning, as well as additional questions about therapy goals, methods, risks, and the therapeutic alliance. For a detailed description of the FPQR, see Saarni et al. (2023).

**Table S2.** Primary symptom measures in different treatments

| Treatment | | Primary measure | |  |
| --- | --- | --- | --- | --- |
| Guided iCBT program | |  | |  |
|  | Depression |  | PHQ-9 (Kroenke et al., 2001); BDI-21^†^(Beck et al., 1961) | |
|  | Generalized anxiety |  | GAD-7 (Spitzer et al., 2006) | |
|  | Panic disorder |  | PDSS-SR (Furukawa et al., 2009) | |
|  | Social anxiety^‡^ |  | SPIN (Connor et al., 2000) | |
|  | Obsessive-compulsive disorder |  | OCI-R (Foa et al., 2002) | |
|  | Harmful use of alcohol |  | AUDIT (Saunders et al., 1993) | |
|  | Bulimia |  | EDE-Q (Fairburn and Beglin, 2011) | |
|  | Bipolar disorder |  | PHQ-9 (Kroenke et al., 2001); BDI-21^†^(Beck et al., 1961) | |
|  | Insomnia |  | ISI (Bastien et al., 2001) | |
|  | Unexplained somatic symptoms |  | Brief IPQ (Broadbent et al., 2006) | |
| ≤20-session psychotherapy | |  | CORE-10^§^ (Barkham et al., 2013); YP-CORE^¶^ (Twigg et al., 2009) | |
| ^†^ BDI-21 used until May 2019  ^‡^ Both adolescent and adult version  ^§^ For adults  ^¶^ For adolescents | | | | |

### Testing assumption of proportional hazards

| **Table S3.**  Proportional hazard test for prior psychological treatment (time-dependent covariate) and entry into therapist-guided iCBT or ≤20-session psychotherapy | | | | | | | |
| --- | --- | --- | --- | --- | --- | --- | --- |
|  | Therapist-guided iCBT | | |  | ≤20-session psychotherapy | | |
| Time dependent covariate | *χ*^2^ | df | p |  | *χ*^2^ | df | p |
| Long-term psychotherapy | 0.06 | 1 | 0.807 |  | 0.42 | 1 | 0.516 |
| ≤20-session psychotherapy | 3.16 | 1 | 0.075 |  |  |  |  |
| Therapist-guided iCBT | - |  |  |  | 0.34 | 1 | 0.558 |
| Adjusted for age, sex, first purchase of psychotropic drugs, first purchase of paracetamol, first psychiatric diagnosis before any treatment studied and treatment interaction with another psychological treatment | | | | | | | |
|  |  |  |  |  |  |  |  |

*
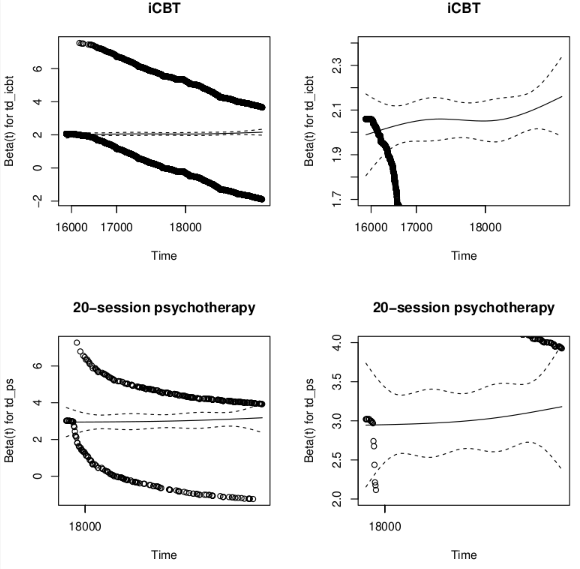
*

**Figure S1.** Cox.zph function plotted for long-term psychotherapy entry. Solid line marking the estimated effect of time-dependent ≤20-session psychotherapy (upper row of panels) or therapist-guided iCBT (lower row) along-term with 95% confidence intervals marked by dotted lines. Time 18000 refers to date 14^th^ of April 2019.


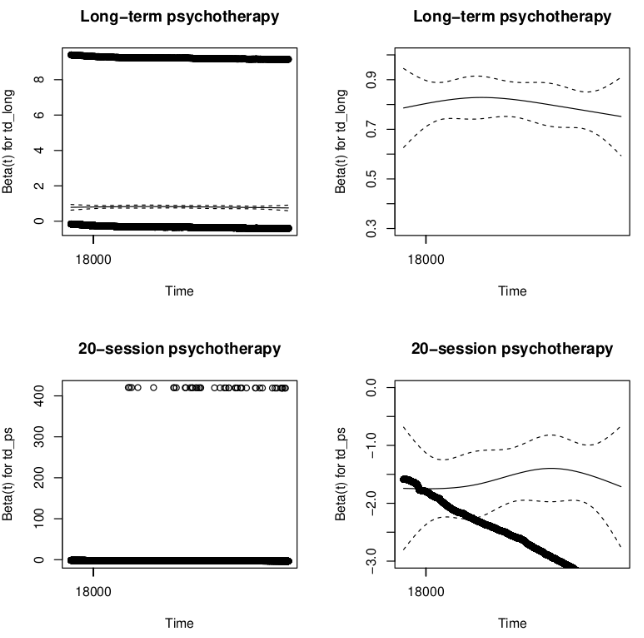


**Figure S2.** Cox.zph function plotted for therapist-guided iCBT entry. Solid line marking the estimated effect of long-term (upper row of panels) or ≤20-session psychotherapy (lower row) along-term with 95% confidence intervals marked by dotted lines. Time 18000 refers to date 14^th^ of April 2019.


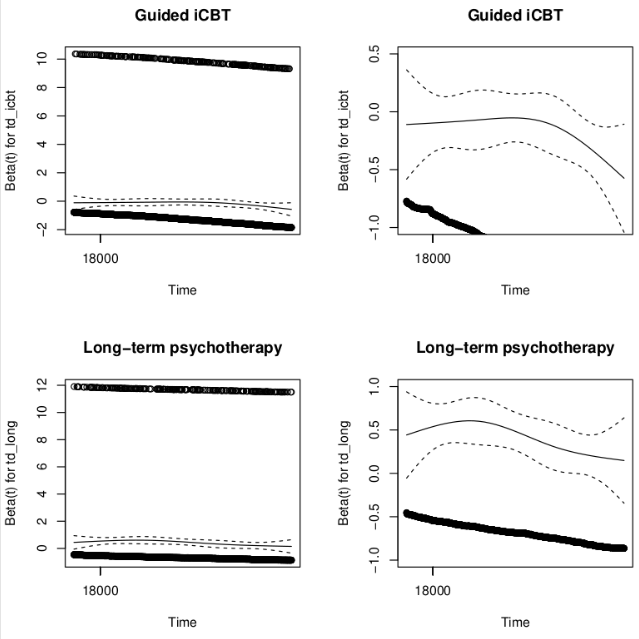


**Figure S3.** Cox.zph function plotted for ≤20-session psychotherapy entry as outcome. Solid line marking the estimated effect of therapist-guided iCBT (upper row of panels) or long-term psychotherapy (lower row) along-term with 95% confidence intervals marked by dotted lines. Time 18000 refers to date 14^th^ of April 2019.

### Consideration of missing symptom change data

In this study, our primary focus was on the real-world effectiveness of the treatments patients actually received, rather than estimating treatments counterfactual efficacy. Using multiple imputation for missing symptom scores in the initial treatment phase would essentially address the counterfactual question: *“What would the hazard ratio for subsequent treatment entry be if every patient had completed the entire initial treatment?”* This approach estimates the treatment’s potential efficacy under ideal, but unrealistic, conditions.

As a sensitivity analysis, we applied multiple imputation to address missing symptom scores in therapist-guided iCBT and ≤20-session psychotherapy including both completely missing values and last observations prior to treatment end. Missing standardized symptom change (as described in the Methods, Covariates section) was imputed using a set of predictors: entry dates for guided iCBT, ≤20-session psychotherapy, and long-term psychotherapy, along with all covariates from the fully adjusted models (age, sex, first purchase of psychotropic drugs, first purchase of paracetamol, and onset of first psychiatric diagnosis in public specialty care before any treatment studied). In addition, we included baseline symptom scores for therapist-guided iCBT and≤20-session psychotherapy, as well as the Nelson–Aalen estimator representing the cumulative baseline hazard for each outcome treatment entry.

Imputation was performed using predictive mean matching with the R-package mice (version 3.17; Buuren and Groothuis-Oudshoorn, 2011). Results from Cox proportional hazards regression analyses for entry into long-term psychotherapy, therapist-guided iCBT, and ≤20-session psychotherapy with imputed symptom change are presented in Supplementary Tables S8, S11 and S15.

### COVID-19 and its impact on treatment delivery

COVID-19 significantly affected the delivery of psychological treatments worldwide. In Finland, face-to-face psychotherapy sessions largely transitioned to video-based formats during the pandemic. However, remote video sessions were already in use prior to 2020, albeit not as widely. These remotely delivered face-to-face psychotherapy sessions are considered as a part of the ≤20-session psychotherapy and long-term psychotherapy in our dataset.

We considered the potential impact of COVID-19 related changes on our main analyses. The Cox proportional hazards model, being semi-parametric, inherently accounts for the changes in base rates over time. The proportional hazards assumption withstood with all the analyzed outcomes and treatments, suggesting that the relative effects of prior treatments remained stable throughout the follow-up period (relative to the varying base rate; see Figure 2, 3.3 Proportional hazard assumptions and Supplement table S3). Therefore, no time-dependent adjustments were required to account for changes in hazard over time, including those specifically related to the COVID-19 period, as these were contained in the base-rate part of the model.

As a sensitivity analysis, we included a time-dependent covariate marking the COVID-19 period. This covariate was activated on March 16, 2020, when a state of emergency was declared in Finland, and remained active until the end of the follow-up (June 30, 2021). These analyses confirmed that the pandemic did not significantly affect hazards ratios: interactions between prior treatment covariates and the COVID-19 time-dependent covariate were non-significant across all outcomes studied (Supplement Table S11).

It is arguably one the great advances of the Cox’s model that in these cases “it is possible to estimate the effect parameter(s) […] without any consideration of the full hazard function”: <https://en.wikipedia.org/wiki/Proportional_hazards_model#Introduction>

| **Table S4.** Cox proportional hazards regression analysis for entry into long-term psychotherapy, therapist-guided iCBT and ≤20-session psychotherapy; interaction between covariate treatment and onset of Covid-19 | | | | | | | | | |
| --- | --- | --- | --- | --- | --- | --- | --- | --- | --- |
|  | Long-term psychotherapy | |  | iCBT |  |  | ≤20-session psychotherapy |  |  |
| Covariate | HR (95% CI)^1^ | p |  | HR (95% CI)^2^ | p |  | HR (95% CI)^3^ | p |  |
| iCBT^1^ | 3.65 (3.33–4.01) | <0.001 |  | - | - |  | 0.39 (0.31–0.49) | <0.001 |  |
| ≤20-session psychotherapy^1^ | 5.04 (3.86–6.58) | <0.001 |  | 0.06 (0.02–0.15) | <0.001 |  | - | - |  |
| Long-term psychotherapy^1^ | - | - |  | 0.95 (0.88–1.03) | 0.195 |  | 1.03 (0.83–1.28) | 0.784 |  |
| Interaction of iCBT^1^ x Covid-19^2^ | 1.05 (0.94–1.18) | 0.395 |  | - | - |  | 0.95 (0.72–1.26) | 0.713 |  |
| Interaction of ≤20-session psychotherapy^1^ x Covid-19^2^ | 1.15 (0.85–1.55) | 0.376 |  | 2.25 (0.80–6.29) | 0.123 |  | - | - |  |
| Interaction of long-term psychotherapy^1^ x Covid-19^2^ | - | - |  | 1.02 (0.93–1.12) | 0.732 |  | 0.92 (0.69–1.22) | 0.551 |  |
| *HR*; hazard ratio, CI;Confidence Interval  Adjusted for age, sex, first purchase of psychotropic drugs, first purchase of paracetamol, first psychiatric diagnosis set in public specialty care before any treatment studied  ^1^Time-dependent covariate  ^2^ Time dependent covariate with onset on 16th March 2020 (date when a state of emergency was declared in Finland over coronavirus outbreak) | | | | | | | | | |

### Sensitive analysis for entry into long-term psychotherapy

| **Table S5.** Cox proportional hazards regression analysis for entry into long-term psychotherapy | | | | | | | | |  |
| --- | --- | --- | --- | --- | --- | --- | --- | --- | --- |
| Covariate | Crude hazard ratio  (95% CI) | p | Age- and sex-adjusted hazard ratio  (95% CI)* | p | Age, sex, diagnosis & drugs adjusted  hazard ratio (95% CI)** | p | All covariates and treatment interaction adjusted hazard ratio (95% CI)^1^ | p | |
| iCBT^2^ | 7.86 (7.48–8.26) | <0.001 | 8.01 (7.78–8.58) | <0.001 | 3.25 (3.08–3.44) | <0.001 | 4.08 (3.81–4.37) | <0.001 | |
| ≤20-session psychotherapy^2^ | 20.50(16.39–25.64) | <0.001 | 21.30 (17.06–26.70) | <0.001 | 16.10 (12.49.–20.85) | <0.001 | 8.94 (7.79–10.26) | <0.001 | |
| iCBT x ≤20-session therapy |  |  |  |  |  |  | 0.14 (0.09–0.22) | <0.001 | |
| Age^3^ |  |  |  |  |  |  | 0.97 (0.97–0.97) | <0.001 | |
| Male (ref: female) |  |  |  |  |  |  | 0.48 (0.45–0.52) | <0.001 | |
| First purchase of psychotropic drugs^2^ |  |  |  |  |  |  | 3.85 (3.56–4.16) | <0.001 | |
| First psychiatric diagnosis^2,4^ |  |  |  |  |  |  | 1.28 (1.19–1.38) | <0.001 | |
| First purchase of paracetamol^2^ |  |  |  |  |  |  | 0.97 (0.91–1.03) | 0.266 | |
| *Adjusted for age and sex  ** Adjusted for age, sex, first purchase of psychotropic drugs, first purchase of paracetamol and first psychiatric diagnosis set in public specialty care before any treatment studied  ^1^Follow-up 322,011 person years from Aug 2018 to June 2021, during which all psychological treatments were available  ^2^ Time-dependent covariate  ^3^Patient’s age at the time of first record in the HUS-iCBT register/FPQR or for controls, age at the time of index patient’s first record  ^4^First psychiatric diagnosis set in public specialty care before any treatment studied | | | | | | | | |  |

###

| **Table S6.** Cox proportional hazards regression results for entry into long-term psychotherapy with interaction between prior psychological treatment and first purchase of psychotropic drugs | | | |
| --- | --- | --- | --- |
|  | Fully adjusted model with interaction of guided iCBT and psychotropic drugs |  | Fully adjusted model with interaction of  ≤20-session psychotherapy and psychotropic drugs |
| Covariate | HR (95% CI)^1^ |  | HR (95% CI)^1^ |
| Guided iCBT^2^ | 13.10 (11.40–15.08)*** |  | 3.79 (3.54–4.05)*** |
| ≤20-session psychotherapy^2^ | 5.57 (4.91–6.33)*** |  | 22.50 (18.42–27.50)*** |
| First purchase of psychotropic drugs^2^ | 5.54 (5.07–6.05)*** |  | 4.35 (4.02–4.72)*** |
| iCBT^2^ x psychotropic drugs^2^ | 0.24 (0.21–0.28)*** |  | - |
| ≤20-session psychotherapy^2^ x psychotropic drugs^2^ | - |  | 0.16 (0.12–0.20)*** |
| Age^3^ | 0.97 (0.97–0.97)*** |  | 0.97 (0.97–0.97)*** |
| Male (ref:female) | 0.48 (0.45–0.52)*** |  | 0.48 (0.45–0.52)*** |
| First psychiatric diagnosis^2,4^ | 1.22 (1.15–1.33)*** |  | 1.27 (1.18–1.36)*** |
| First purchase of paracetamol^2^ | 0.95 (0.89–1.00)* |  | 0.98 (0.92–1.03) |
| ^1^HR=Hazard Ratio, CI=Confidence Interval  ^2^ Time-dependent covariate  ^3^Patient’s age at the time of first record in the HUS-iCBT register/FPQR or for controls, age at the time of index patient’s first record  ^4^First psychiatric diagnosis set in public specialty care before any treatment studied  *p<0.05, **p<0.01, ***p<0.001 | | | |
|  |  |  |  |
|  |  |  |  |
|  |  |  |  |

| **Table S7.** Cox proportional hazards regression results for entry into long-term psychotherapy including interaction between prior psychological treatment and its symptom change | | | | | |
| --- | --- | --- | --- | --- | --- |
|  |  | Crude hazard ratio  (95% CI) | p | All covariates and interaction adjusted hazard ratio (95% CI)^1^ | p |
| Model for guided iCBT | |  |  |  |  |
|  | iCBT^2^ | 7.77 (7.35–8.22) | <0.001 | 3.75 (3.51–4.01) | <0.001 |
|  | iCBT x symptom change^2,3^ | 0.99 (0.96–1.04) | 0.966 | 1.02 (0.98–1.07) | 0.322 |
|  | | | | | |
| Model for ≤20-session psychotherapy | |  |  |  |  |
|  | ≤20-session psychotherapy^2^ | 6.51 (5.72–7.41) | <0.001 | 5.50 (4.83–6.26) | <0.001 |
|  | ≤20-session psychotherapy x symptom change^2,3^ | 0.78 (0.68–0.90) | <0.001 | 0.81 (0.71–0.92) | <0.01 |
| HR=Hazard Ratio, CI=Confidence Interval  ^1^Adjusted for other prior psychological treatment and its symptom change (iCBT or ≤20-session psychotherapy), age, sex, first purchase of psychotropic drugs, first purchase of paracetamol and first psychiatric diagnosis set in public specialty care before any treatment studied  ^2^ Time-dependent covariate  ^3^Calculated as the difference between first and last target symptom score and standardized within each treatment (see Table S1) | | | | | |

| **Table S8.** Cox proportional hazards regression results for entry into long-term psychotherapy including interaction between prior psychological treatment and its imputed symptom change | | | | | |
| --- | --- | --- | --- | --- | --- |
|  |  | Crude hazard ratio  (95% CI) | p | All covariates and interaction adjusted hazard ratio (95% CI)^1^ | p |
| Model for guided iCBT | |  |  |  |  |
|  | iCBT^2^ | 7.75 (7.33–8.20) | <0.001 | 3.73 (3.49–3.99) | <0.001 |
|  | iCBT x symptom change^2,3^ | 0.92 (0.87–0.97) | <0.01 | 0.91 (0.86–0.96) | <0.001 |
|  | | | | | |
| Model for ≤20-session psychotherapy | |  |  |  |  |
|  | ≤20-session psychotherapy^2^ | 6.47 (5.67–7.37) | <0.001 | 5.43 (4.77–6.19) | <0.001 |
|  | ≤20-session psychotherapy x symptom change^2,3^ | 0.78 (0.67–0.90) | <0.001 | 0.82 (0.72–0.93) | <0.01 |
| HR=Hazard Ratio, CI=Confidence Interval  ^1^Adjusted for each other, age, sex, first purchase of psychotropic drugs, first purchase of paracetamol and first psychiatric diagnosis set in public specialty care before any treatment studied  ^2^ Time-dependent covariate  ^3^Imputed standardized symptom change (see Supplementary section 4) | | | | | |

### Sensitive analysis for entry into therapist-guided iCBT

| **Table S9.** Cox proportional hazards regression analysis for entry into therapist-guided iCBT | | | | | | | | |
| --- | --- | --- | --- | --- | --- | --- | --- | --- |
| Covariate | Crude hazard ratio  (95% CI) | p | Age and sex adjusted hazard ratio  (95% CI)^1^ | p | Age, sex, diagnosis & drugs adjusted  hazard ratio (95% CI)^2^ | p | All covariates and treatment interaction adjusted hazard ratio (95% CI)^3^ | p |
| Long-term psychotherapy^4^ | 2.17 (2.09–2.26) | <0.001 | 2.18 (2.09–2.27) | <0.001 | 0.86 (0.83–0.90) | <0.001 | 0.96 (0.92–1.00) | 0.087 |
| ≤20-session psychotherapy^4^ | 0.20 (0.15–0.27) | <0.001 | 0.21 (0.15–0.28) | <0.001 | 0.12 (0.09–0.16) | <0.001 | 0.12 (0.09–0.16) | <0.001 |
| Long-term x ≤20-session psychotherapy^4^ |  |  |  |  |  |  | 0.97 (0.45–2.09) | 0.934 |
| Age^5^ |  |  |  |  |  |  | 0.99 (0.99–0.99) | <0.001 |
| Male (ref: female) |  |  |  |  |  |  | 1.11 (1.07–1.14) | <0.001 |
| First purchase of psychotropic drugs^4^ |  |  |  |  |  |  | 6.00 (5.76–6.24) | <0.001 |
| First psychiatric diagnosis^4,6^ |  |  |  |  |  |  | 2.47 (2.39-2.55) | <0.001 |
| First purchase of paracetamol^4^ |  |  |  |  |  |  | 0.99 (0.96–1.02) | 0.538 |
| ^1^Adjusted for age and sex  ^2^ Adjusted for age, sex, first purchase of psychotropic drugs, first purchase of paracetamol and onset of first psychiatric diagnosis set in public specialty care before any treatment studied  ^3^ Follow-up 311,543 person years from Aug 2018 to June 2021, during which all psychological treatments were available  ^4^ Time-dependent covariate  ^5^Patient’s age at the time of first record in the HUS-iCBT register/FPQR or for controls, age at the time of index patient’s first record  ^6^ First psychiatric diagnosis set in public specialty care before any treatment studied | | | | | | | | |

| **Table S10.** Cox proportional hazards regression results for entry into therapist-guided iCBT including interaction between prior ≤20-session psychotherapy and its symptom change | | | | |
| --- | --- | --- | --- | --- |
|  | Crude hazard ratio  (95% CI) | p | All covariates and interaction adjusted hazard ratio (95% CI)^1^ | p |
| ≤20-session psychotherapy^2^ | 0.19 (0.14–0.26) | <0.001 | 0.11 (0.08–0.15) | <0.001 |
| ≤20-session psychotherapy x symptom change^2,3^ | 0.64 (0.46–0.88) | <0.01 | 0.65 (0.47–0.89) | <0.01 |
| HR=Hazard Ratio, CI=Confidence Interval  ^1^Adjusted for age, sex, long-term psychotherapy, first purchase of psychotropic drugs, first purchase of paracetamol and first psychiatric diagnosis set in public specialty care before any treatment studied  ^2^ Time-dependent covariate  ^3^Calculated as the difference between first and last observed target symptom score and standardized within each treatment (see Table S1) | | | | |

| **Table S11**. Cox proportional hazards regression results for entry into therapist-guided iCBT including interaction between prior ≤20-session psychotherapy and its imputed symptom change | | | | |
| --- | --- | --- | --- | --- |
|  | Crude hazard ratio  (95% CI) | p | All covariates and interaction adjusted hazard ratio (95% CI)^1^ | p |
| ≤20-session psychotherapy^2^ | 0.19 (0.14–0.27) | <0.001 | 0.11 (0.08–0.15) | <0.001 |
| ≤20-session psychotherapy x symptom change^2,3^ | 0.70 (0.51–0.95) | <0.05 | 0.70 (0.52–0.95) | <0.05 |
| HR=Hazard Ratio, CI=Confidence Interval  ^1^Adjusted for age, sex, long-term psychotherapy, first purchase of psychotropic drugs, first purchase of paracetamol and first psychiatric diagnosis set in public specialty care before any treatment studied  ^2^ Time-dependent covariate  ^3^Imputed standardized symptom change (see Supplementary section 4) | | | | |

### Sensitive analysis for entry into ≤20-session psychotherapy

| **Table S12.** Cox proportional hazards regression analysis for entry into up to ≤20-session psychotherapy | | | | | | | | |  |
| --- | --- | --- | --- | --- | --- | --- | --- | --- | --- |
| Covariate | Crude hazard ratio (95% CI) | p | Age- and sex- adjusted hazard ratio (95% CI)^1^ | p | Age-, sex-, diagnosis- and drugs-adjusted hazard ratio (95% CI)^2^ | p | All covariates and treatment interaction adjusted hazard ratio (95% CI)^3^ | p | |
| iCBT^2^ | 0.85 (0.75–0.97) | <0.05 | 0.86 (0.76–0.99) | <0.05 | 0.37 (0.32–0.43) | <0.001 | 0.41 (0.35–0.47) | <0.001 | |
| Long-term psychotherapy^4^ | 1.51 (1.32–1.74) | <0.001 | 1.47 (1.28–1.69) | <0.001 | 0.94 (0.81–1.08) | 0.359 | 1.08 (0.93–1.25) | 0.332 | |
| iCBT x long-term therapy^4^ |  |  |  |  |  |  | 0.53 (0.34–0.81) | <0.05 | |
| Age^5^ |  |  |  |  |  |  | 1.02 (1.02–1.02) | <0.001 | |
| Male (ref: female) |  |  |  |  |  |  | 0.67 (0.61–0.74) | <0.001 | |
| First purchase of psychotropic drugs^4^ |  |  |  |  |  |  | 0.93 (0.85–1.02) | 0.137 | |
| First psychiatric diagnosis^4,6^ |  |  |  |  |  |  | 6.63 (6.05–7.27) | <0.001 | |
| First purchase of paracetamol^4^ |  |  |  |  |  |  | 0.62 (0.56-0.68) | <0.001 | |
| ^1^Adjusted for age and sex  ^2^Adjusted for age, sex, first purchase of psychotropic drugs, first purchase of paracetamol and onset of first psychiatric diagnosis set in public specialty care before any treatment studied  ^3^Follow-up 132,490 person years from Aug 2018 to June 2021, during which all psychological treatments were available  ^4^ Time-dependent covariate  ^5^Patient’s age at the time of first record in the HUS-iCBT register/FPQR or for controls, age at the time of index patient’s first record  ^6^First psychiatric diagnosis set in public specialty care before any treatment studied | | | | | | | | |  |

| **Table S13.** Cox proportional hazards regression results for entry into up to ≤20-session psychotherapy: interaction between age at registry onset and first drug purchase (either paracetamol or psychotropics) | | | | | | | | |
| --- | --- | --- | --- | --- | --- | --- | --- | --- |
|  | Paracetamol x age interaction model |  | Fully adjusted model with  paracetamol x age interaction |  | Psychotropics x age interaction model |  | Fully adjusted model with psychotropics x age interaction |  |
| Covariate | HR (95% CI)^1^ |  | HR (95% CI)^1^ |  | HR (95% CI)^1^ |  | HR (95% CI)^1^ |  |
| First purchase of paracetamol^2^ | 1.134 (0.88–1.46) |  | 0.92 (0.72–1.19) |  | - |  | 0.63 (0.57-0.69)*** |  |
| First purchase of psychotropic drugs^2^ | - |  | 0.95 (0.86–1.04 |  | 2.51 (1.99–3.16)*** |  | 1.36 (1.08–1.72)* |  |
| Age^3^ | 1.02 (1.01–1.02)*** |  | 1.02 (1.02–1.03)*** |  | 1.02 (1.01–1.02)*** |  | 1.02 (1.02–1.03)*** |  |
| Age x first drug purchase | 0.99 (0.99–1.00)*** |  | 0.99 (0.98–1.00)*** |  | 0.98 (0.98–0.99)*** |  | 0.99 (0.98-1.00)*** |  |
| Long-term psychotherapy^2^ |  |  | 0.97 (0.84–1.12) |  |  |  | 0.97 (0.84–1.11) |  |
| iCBT^2^ |  |  | 0.37 (0.32–0.43)*** |  |  |  | 0.37 (0.32-0.43)*** |  |
| Male (ref:female) |  |  | 0.67 (0.61–0.74)*** |  |  |  | 0.67 (0.61–0.74)*** |  |
| First psychiatric diagnosis^2,4^ |  |  | 6.67 (6.09–7.31)*** |  |  |  | 6.70 (6.11–7.34)*** |  |
| ^1^HR=Hazard Ratio, CI=Confidence Interval  ^2^ Time-dependent covariate  ^3^ Patient’s age at the time of first record in the HUS-iCBT register/FPQR or for controls, age at the time of index patient’s first record  ^4^ First psychiatric diagnosis set in public specialty care before any treatment studied  *p<0.05, **p<0.01, ***p<0.001 | | | | | | | | |

| **Table S14.** Cox proportional hazards regression results for entry into ≤20-session psychotherapy including interaction between prior therapist-guided iCBT and its symptom change | | | | |
| --- | --- | --- | --- | --- |
| Covariate | Crude hazard ratio  (95% CI)^1^ | p | All covariates and interaction adjusted hazard ratio (95% CI)^1,2^ | p |
| Therapist-guided iCBT^3^ | 0.75 (0.65–0.87) | <0.001 | 0.33 (0.28–0.38) | <0.001 |
| iCBT x symptom change^3,4^ | 0.49 (0.43–0.57) | <0.001 | 0.51 (0.44–0.59) | <0.001 |
| ^1^ Follow-up 132,490 person years from Aug 2018 to June 2021, during which all psychological treatments were available  ^2^Adjusted for age, sex, long-term psychotherapy, first purchase of psychotropic drugs, first purchase of paracetamol and onset of first psychiatric diagnosis set in public specialty care before any treatment studied  ^3^ Time dependent covariate  ^4^Calculated as the difference between first and last observed target symptom score and standardized within each treatment (see Table S1) | | | | |

| **Table S15.** Cox proportional hazards regression results for entry into ≤20-session psychotherapy including interaction between prior therapist-guided iCBT and its imputed symptom change | | | | |
| --- | --- | --- | --- | --- |
| Covariate | Crude hazard ratio  (95% CI)^1^ | p | All covariates and interaction adjusted hazard ratio (95% CI)^1^ | p |
| Therapist-guided iCBT^2^ | 0.78 (0.68–0.90) | <0.001 | 0.34 (0.29–0.40) | <0.001 |
| iCBT x symptom change^2,3^ | 0.65 (0.57–0.74) | <0.001 | 0.65 (0.57–0.74) | <0.001 |
| ^1^Adjusted for age, sex, long-term psychotherapy, first purchase of psychotropic drugs, first purchase of paracetamol and onset of first psychiatric diagnosis set in public specialty care before any treatment studied  ^2^ Time dependent covariate  ^3^ Imputed standardized symptom change (see Supplementary section 4) | | | | |

**References**

Barkham, M., Bewick, B., Mullin, T., Gilbody, S., Connell, J., Cahill, J., Mellor-Clark, J., Richards, D., Unsworth, G., Evans, C., 2013. The CORE-10: A short measure of psychological distress for routine use in the psychological therapies. Couns. Psychother. Res. 13, 3–13. https://doi.org/10.1080/14733145.2012.729069

Barrio-Martínez, S., Ruiz-Rodríguez, P., Medrano, L.A., Priede, A., Muñoz-Navarro, R., Moriana, J.A., Carpallo-González, M., Prieto-Vila, M., Cano-Vindel, A., González-Blanch, C., 2024. Effect of Reliable Recovery on Health Care Costs and Productivity Losses in Emotional Disorders. Behav. Ther. 55, 585–594. https://doi.org/10.1016/j.beth.2023.08.012

Bastien, C.H., Vallières, A., Morin, C.M., 2001. Validation of the Insomnia Severity Index as an outcome measure for insomnia research. Sleep Med. 2, 297–307. https://doi.org/10.1016/s1389-9457(00)00065-4

Beatty, L., Binnion, C., 2016. A Systematic Review of Predictors of, and Reasons for, Adherence to Online Psychological Interventions. Int. J. Behav. Med. 23, 776–794. https://doi.org/10.1007/s12529-016-9556-9

Beck, A.T., Ward, C.H., Mendelson, M., Mock, J., Erbaugh, J., 1961. An inventory for measuring depression. Arch. Gen. Psychiatry 4, 561–571. https://doi.org/10.1001/archpsyc.1961.01710120031004

Broadbent, E., Petrie, K.J., Main, J., Weinman, J., 2006. The brief illness perception questionnaire. J. Psychosom. Res. 60, 631–637. https://doi.org/10.1016/j.jpsychores.2005.10.020

Buckman, J.E.J., Underwood, A., Clarke, K., Saunders, R., Hollon, S.D., Fearon, P., Pilling, S., 2018. Risk factors for relapse and recurrence of depression in adults and how they operate: A four-phase systematic review and meta-synthesis. Clin. Psychol. Rev. 64, 13–38. https://doi.org/10.1016/j.cpr.2018.07.005

Buuren, S. van, Groothuis-Oudshoorn, K., 2011. mice: Multivariate Imputation by Chained Equations in R. J. Stat. Softw. 45, 1–67. https://doi.org/10.18637/jss.v045.i03

Catarino, A., Bateup, S., Tablan, V., Innes, K., Freer, S., Richards, A., Stott, R., Hollon, S.D., Chamberlain, S.R., Hayes, A., Blackwell, A.D., 2018. Demographic and clinical predictors of response to internet-enabled cognitive–behavioural therapy for depression and anxiety. BJPsych Open 4, 411–418. https://doi.org/10.1192/bjo.2018.57

Connor, K.M., Davidson, J.R., Churchill, L.E., Sherwood, A., Foa, E., Weisler, R.H., 2000. Psychometric properties of the Social Phobia Inventory (SPIN). New self-rating scale. Br. J. Psychiatry J. Ment. Sci. 176, 379–386. https://doi.org/10.1192/bjp.176.4.379

Cuijpers, P., Karyotaki, E., Eckshtain, D., Ng, M.Y., Corteselli, K.A., Noma, H., Quero, S., Weisz, J.R., 2020. Psychotherapy for Depression Across Different Age Groups: A Systematic Review and Meta-analysis. JAMA Psychiatry 77, 694–702. https://doi.org/10.1001/jamapsychiatry.2020.0164

Delgadillo, J., Rhodes, L., Moreea, O., McMillan, D., Gilbody, S., Leach, C., Lucock, M., Lutz, W., Ali, S., 2018. Relapse and Recurrence of Common Mental Health Problems after Low Intensity Cognitive Behavioural Therapy: The WYLOW Longitudinal Cohort Study. Psychother. Psychosom. 87, 116–117. https://doi.org/10.1159/000485386

Fairburn, C.G., Beglin, S.J., 2011. Eating Disorder Examination Questionnaire. https://doi.org/10.1037/t03974-000

Foa, E.B., Huppert, J.D., Leiberg, S., Langner, R., Kichic, R., Hajcak, G., Salkovskis, P.M., 2002. The Obsessive-Compulsive Inventory: Development and validation of a short version. Psychol. Assess. 14, 485–496. https://doi.org/10.1037/1040-3590.14.4.485

Furukawa, T.A., Shear, M.K., Barlow, D.H., Gorman, J.M., Woods, S.W., Money, R., Etschel, E., Engel, R.R., Leucht, S., 2009. Evidence-based Guidelines for Interpretation of the Panic Disorder Severity Scale. Depress. Anxiety 26, 922–929. https://doi.org/10.1002/da.20532

HUS, 2025. Nettiterapian tuloksellisuus | Mielenterveystalo.fi [WWW Document]. URL https://www.mielenterveystalo.fi/fi/nettiterapian-tuloksellisuus (accessed 3.20.25).

Karyotaki, E., Ebert, D.D., Donkin, L., Riper, H., Twisk, J., Burger, S., Rozental, A., Lange, A., Williams, A.D., Zarski, A.C., Geraedts, A., van Straten, A., Kleiboer, A., Meyer, B., Ünlü Ince, B.B., Buntrock, C., Lehr, D., Snoek, F.J., Andrews, G., Andersson, G., Choi, I., Ruwaard, J., Klein, J.P., Newby, J.M., Schröder, J., Laferton, J.A.C., Van Bastelaar, K., Imamura, K., Vernmark, K., Boß, L., Sheeber, L.B., Kivi, M., Berking, M., Titov, N., Carlbring, P., Johansson, R., Kenter, R., Perini, S., Moritz, S., Nobis, S., Berger, T., Kaldo, V., Forsell, Y., Lindefors, N., Kraepelien, M., Björkelund, C., Kawakami, N., Cuijpers, P., 2018. Do guided internet-based interventions result in clinically relevant changes for patients with depression? An individual participant data meta-analysis. Clin. Psychol. Rev. 63, 80–92. https://doi.org/10.1016/j.cpr.2018.06.007

Karyotaki, E., Kleiboer, A., Smit, F., Turner, D.T., Pastor, A.M., Andersson, G., Berger, T., Botella, C., Breton, J.M., Carlbring, P., Christensen, H., Graaf, E. de, Griffiths, K., Donker, T., Farrer, L., Huibers, M.J.H., Lenndin, J., Mackinnon, A., Meyer, B., Moritz, S., Riper, H., Spek, V., Vernmark, K., Cuijpers, P., 2015. Predictors of treatment dropout in self-guided web-based interventions for depression: an ‘individual patient data’ meta-analysis. Psychol. Med. 45, 2717–2726. https://doi.org/10.1017/S0033291715000665

Kessing, L.V., Ziersen, S.C., Caspi, A., Moffitt, T.E., Andersen, P.K., 2023. Lifetime Incidence of Treated Mental Health Disorders and Psychotropic Drug Prescriptions and Associated Socioeconomic Functioning. JAMA Psychiatry 80, 1000–1008. https://doi.org/10.1001/jamapsychiatry.2023.2206

Kroenke, K., Spitzer, R.L., Williams, J.B.W., 2001. The PHQ-9. J. Gen. Intern. Med. 16, 606–613. https://doi.org/10.1046/j.1525-1497.2001.016009606.x

Leppänen, H., Kampman, O., Autio, R., Karolaakso, T., Näppilä, T., Rissanen, P., Pirkola, S., 2022. Socioeconomic factors and use of psychotherapy in common mental disorders predisposing to disability pension. BMC Health Serv. Res. 22, 983. https://doi.org/10.1186/s12913-022-08389-1

Lorimer, B., Kellett, S., Giesemann, J., Lutz, W., Delgadillo, J., 2024. An investigation of treatment return after psychological therapy for depression and anxiety. Behav. Cogn. Psychother. 52, 149–162. https://doi.org/10.1017/S1352465823000322

Palacios, J.E., Enrique, A., Mooney, O., Farrell, S., Earley, C., Duffy, D., Eilert, N., Harty, S., Timulak, L., Richards, D., 2022. Durability of treatment effects following internet-delivered cognitive behavioural therapy for depression and anxiety delivered within a routine care setting. Clin. Psychol. Psychother. 29, 1768–1777. https://doi.org/10.1002/cpp.2743

Reeder, K., Park, A.L., Chorpita, B.F., 2020. Turning Back to Treatment: The Effect of Attendance and Symptom Outcomes on Subsequent Service Use. Adm. Policy Ment. Health Ment. Health Serv. Res. 47, 641–647. https://doi.org/10.1007/s10488-020-01032-3

Reins, J.A., Buntrock, C., Zimmermann, J., Grund, S., Harrer, M., Lehr, D., Baumeister, H., Weisel, K., Domhardt, M., Imamura, K., Kawakami, N., Spek, V., Nobis, S., Snoek, F., Cuijpers, P., Klein, J.P., Moritz, S., Ebert, D.D., 2020. Efficacy and Moderators of Internet-Based Interventions in Adults with Subthreshold Depression: An Individual Participant Data Meta-Analysis of Randomized Controlled Trials. Psychother. Psychosom. 90, 94–106. https://doi.org/10.1159/000507819

Saarni, S.E., Rosenström, T., Stenberg, J.-H., Plattonen, A., Holi, M., Ekelund, J., Granö, N., Komsi, N., Saarni, S.I., 2023. Finnish Psychotherapy Quality Register: rationale, development, and baseline results. Nord. J. Psychiatry 77, 455–466. https://doi.org/10.1080/08039488.2022.2150788

Saunders, J.B., Aasland, O.G., Babor, T.F., de la Fuente, J.R., Grant, M., 1993. Development of the Alcohol Use Disorders Identification Test (AUDIT): WHO Collaborative Project on Early Detection of Persons with Harmful Alcohol Consumption--II. Addict. Abingdon Engl. 88, 791–804. https://doi.org/10.1111/j.1360-0443.1993.tb02093.x

Selinheimo, S., Gluschkoff, K., Kausto, J., Turunen, J., Väänänen, A., 2023. Sociodemographic Factors as Predictors of the Duration of Long-term Psychotherapy: Evidence from a Finnish Nationwide Register Study. Adm. Policy Ment. Health Ment. Health Serv. Res. https://doi.org/10.1007/s10488-023-01305-7

Smith, O.R.F., Aarø, L.E., Knapstad, M., 2023. The Importance of Symptom Reduction for Functional Improvement after Cognitive Behavioral Therapy for Anxiety and Depression: A Causal Mediation Analysis. Psychother. Psychosom. 92, 193–202. https://doi.org/10.1159/000530650

Smith, O.R.F., Clark, D.M., Hensing, G., Layard, R., Knapstad, M., 2025. Cost–benefit of IAPT Norway and effects on work-related outcomes and health care utilization: results from a randomized controlled trial using registry-based data. Psychol. Med. 55, e86. https://doi.org/10.1017/S003329172500025X

Spitzer, R.L., Kroenke, K., Williams, J.B.W., Löwe, B., 2006. A brief measure for assessing generalized anxiety disorder: the GAD-7. Arch. Intern. Med. 166, 1092–1097. https://doi.org/10.1001/archinte.166.10.1092

Stubbs, B., Vancampfort, D., Veronese, N., Thompson, T., Fornaro, M., Schofield, P., Solmi, M., Mugisha, J., Carvalho, A.F., Koyanagi, A., 2017. Depression and pain: primary data and meta-analysis among 237 952 people across 47 low- and middle-income countries. Psychol. Med. 47, 2906–2917. https://doi.org/10.1017/S0033291717001477

Suokas, K., Niemi, R., Gutvilig, M., McGrath, J.J., Komulainen, K., Suvisaari, J., Elovainio, M., Lumme, S., Pirkola, S., Hakulinen, C., 2025. Lifetime incidence and age of onset of mental disorders, and 12-month service utilization in primary and secondary care: a Finnish nationwide registry study. Epidemiol. Psychiatr. Sci. 34, e31. https://doi.org/10.1017/S2045796025100061

Twigg, E., Barkham, M., Bewick, B.M., Mulhern, B., Connell, J., Cooper, M., 2009. The Young Person’s CORE: Development of a brief outcome measure for young people. Couns. Psychother. Res. 9, 160–168. https://doi.org/10.1080/14733140902979722

Wojnarowski, C., Firth, N., Finegan, M., Delgadillo, J., 2019. Predictors of depression relapse and recurrence after cognitive behavioural therapy: a systematic review and meta-analysis. Behav. Cogn. Psychother. 47, 514–529. https://doi.org/10.1017/S1352465819000080
